# Exosomal miRNA expression in transplant recipients with EBV-associated post-transplant lymphoproliferative disorder

**DOI:** 10.3389/fimmu.2026.1862392

**Published:** 2026-07-10

**Authors:** Martyna Pociupany, Robert Snoeck, Daan Dierickx, Graciela Andrei

**Affiliations:** 1 Molecular Structural and Translational Virology Research Group, Rega Institute for Medical Research, Department of Microbiology, Immunology and Transplantation, Katholieke Universiteit (KU) Leuven, Leuven, Belgium; 2 Department of Hematology, University Hospitals Leuven, Leuven, Belgium; 3 Laboratory of Experimental Hematology, Department of Oncology, Katholieke Universiteit (KU) Leuven, Leuven, Belgium

**Keywords:** exosomes, hematopoietic stem cell transplantation, HSCT, MicroRNAs, posttransplant lymphoproliferative disorder, PTLD, transplantation

## Abstract

**Background:**

Epstein-Barr Virus (EBV) drives the development of EBV-associated malignancies such as post-transplant lymphoproliferative disorder (PTLD), evading immune detection. However, how EBV is able to evade immune surveillance is not well understood. One theory suggests that modulation of host microRNAs (miRNAs) expression can help escape immune recognition. EBV-encoded miRNAs support tumor cells survival and manipulate expression of host miRNAs. EBV-infected cells package their content into exosomes, which are transferred throughout the body. Tumor and infected cells by packaging their oncogenic or viral cargo into exosomes can spread the disease and infection. Exosomal cargo is being recognized as potential non-invasive biomarker.

**Objectives:**

The aim of this investigation was to analyse the expression of both host- and EBV-encoded miRNAs in exosomes derived from PTLD patients after solid organ transplantation (SOT) or allogeneic hematopoietic stem cell transplantation (HSCT).

**Patients and methods:**

We examined the expression of 28 miRNAs (13 EBV- and 15 host-encoded) from plasma of 23 patients post-HSCT with PTLD (PTLD-HSCT) and 25 post-HSCT patients without PTLD (Non-PTLD-HSCT) at 3 different timepoints (T0 [at the time of HSCT], T1 [3 months post-HSCT] and T2 [6 months post-HSCT]. We also analyzed plasma of 10 solid organ transplant recipients (SOT) with EBV-positive PTLD (PTLD-SOT-EBV(+)) and 8 with EBV-negative PTLD (PTLS-SOT-EBV(-)) and 25 healthy donors.

**Results:**

There was no difference in miRNAs expression between PTLD-HSCT and Non-PTLD-HSCT group. Significant differences in miRNAs expression between PTLD patients (post-SOT and post-HSCT) and healthy donors was observed. miRNAs profile differed between EBV(+) and EBV(-) PTLD patients.

**Conclusions:**

We highlight the importance of an intermediate control group for selecting PTLD-specific biomarker. Future research should focus on detailed characterization of additional components of the exosomal cargo to identify reliable and non-invasive biomarkers that are critical and specific for PTLD development.

## 1 Introduction

Post-transplant lymphoproliferative disorder (PTLD) is a life-threatening complication arising from either solid organ transplantation (SOT) or allogeneic hematopoietic stem cell transplantation (HSCT) (1–4). PTLD is associated with Epstein-Barr virus (EBV) infection, which drives the uncontrolled proliferation of infected B-cells (1–4). EBV is an ubiquitous γ-herpesvirus, establishing lifelong latency without causing clinical symptoms (1, 5, 6). However, in immunocompromised patients, EBV can drive the development of various malignancies like nasopharyngeal carcinoma (NPC), gastric carcinoma (GC), Burkitt Lymphoma (BL), Hodgkin Lymphoma (HL) or PTLD (1, 5, 6). The mechanism by which EBV is able to evade immune detection is still not completely understood (1). One theory suggests that modulation of host microRNAs (miRNAs) expression can facilitate EBV immune escape (1). miRNAs are small, non-coding and highly conserved RNAs regulating various biological processes including gene expression, cell cycle or cell differentiation (6–9). EBV was the first human virus identified to encode viral miRNAs (6–9). EBV expresses 25 pre-miRNAs encoded within 2 regions (BamHI fragment H rightward open reading frame 1 [BHRF1] and BamHI-A rightward transcript [BART]) and around 44 mature miRNAs (5–10). EBV-encoded miRNAs support the survival of tumor cells by inhibiting apoptosis, promoting angiogenesis or metastasis and by modulating the expression of host miRNAs (5–10). Moreover, miRNA dysregulation is often described in hematological malignancies (9). EBV-infected cells package their content such as viral and host-encoded miRNAs into exosomes, which can reach recipient cells and facilitate intercellular communication, contributing to immune evasion and disease progression (11). Exosomes are small (40–100 nm), extracellular vesicles released by normal, tumor or infected cells into body fluids, where they can circulate throughout the body (6, 8, 11). By packaging their oncogenic or viral cargo into exosomes, tumor and infected cells influence recipient cells, contributing to disease progression (6, 8, 11). Exosomes derived from EBV-associated tumors contain oncogenic cargo that exert tumorigenic and immunosuppressive effects on uninfected cells (6, 8, 11). Both viral and host miRNAs are protected from degradation by RNases (ribonucleases) due to their incorporation within exosomal membrane, enabling their stable transport and delivery to recipient cells (11, 12). The diagnostic potential of the exosome cargo is increasingly being recognized as non-invasive biomarkers across different diseases (12). In this study, we aimed to investigate the expression of both host and EBV-encoded miRNAs in HSCT and SOT recipients who developed PTLD and to compare their miRNA expression profile. We analyzed the expression of 28 miRNAs (13 EBV-encoded and 15 host miRNAs) in plasma samples obtained from 23 patients following HSCT who developed PTLD (PTLD-HSCT), 25 patients after HSCT who did not develop PTLD (Non-PTLD-HSCT) at 3 different timepoints (T0 [at the time of the transplant], T1 [3 months post HSCT] and T2 [6 months post HSCT]. In addition, plasma samples from 10 SOT patients, who developed EBV positive PTLD (PTLD-SOT-EBV(+)) and 8 SOT patients developing EBV negative PTLD (PTLS-SOT-EBV(-)) were analyzed at various timepoints. We also included plasma samples from 25 healthy donors as control.

## 2 Methods

### 2.1 Study population

Forty-eight patients who underwent HSCT in the University Hospital of Leuven (UZ Leuven) between November 2008 and June 2018 were enrolled: 23 patients that after HSCT developed PTLD (PTLD-HSCT) and 25 patients that after HSCT did not develop PTLD (Non-PTLD-HSCT). Additionally, 18 patients after SOT, who developed PTLD between February 2016 and December 2023 were enrolled, with 10 patients being EBV-positive (PTLD-SOT-EBV(+)), and 8 being EBV-negative (PTLD-SOT-EBV(-)). Moreover, 25 healthy donors without any diagnosed disease, were included. Demographics and hematological and transplant-related characteristic were retrieved from medical records. HD personal data were anonymous. The Ethics Committee of the UZ/KU Leuven approved the protocol before initiation of the study (S62534 and S69378).

### 2.2 Collection of human plasma

Plasma samples were obtained from 23 PTLD-HSCT and 25 Non-PTLD-HSCT patients at three different time points: at the time of transplantation (T0), 3 months (T1) and 6 months (T2) after HSCT. Plasma from PTLD-SOT-EBV(+) and PTLD-SOT-EBV(-) was obtained at different timepoints (ranging from samples taken at transplantation [T0] to samples taken at 3 months [T1], 6 months [T2] and over 19 months post-SOT [T3]). HD plasma was obtained only once. The samples were kept at -80°C.

### 2.3 Exosomal RNA extraction

RNA from exosomes was isolated from 1 ml of plasma using the Plasma/Serum Exosome Purification and RNA isolation kit (Norgen Biotek, Cat #58300) according to the manufacturer’s instructions. Purified exosomal RNA of all sizes including miRNA, from patients’ samples were tested for amplification of 5S rRNA (housekeeping gene).

### 2.4 Quantitative miRNA RT-qPCR

cDNA was synthesized from each sample using the miRCURY LNA RT Kit (Qiagen) according to the manufacturer’s instructions. The assay is based on a poly(A)-tailing reverse transcription PCR method, in which a poly(A) tail is added to mature miRNAs and other small RNAs, followed by reverse transcription using an oligo(dT) primer containing a universal tag. This enables sensitive and specific LNA-enhanced qPCR detection. Next, qPCR for miRNA detection was performed. miRCury LNA miRNA custom PCR panels (Qiagen) were designed with the use of Qiagen online software, containing specific forward and reverse primers for the 28 candidate miRNAs, one internal reference miRNA (rRNA 5S), UniSP6 and UniSP3 (template control) as well as blank control (**Supplementary Table 1****).** cDNA was diluted 40x with the use of nuclease free water and a 10 μl mixture of 5 μL miRcury LNA Sybr Green (Qiagen), 4 μL diluted cDNA, 0.05 μL of ROX dye and 0.95 μL nuclease-free water per reaction was prepared. qPCR was performed with the use of a Quant Studio7, according to the miRCury LNA miRNA Custom PCR Panels (Qiagen) handbook with 40 amplification cycles. Each sample was analyzed by qPCR in duplicate. The samples with CT values >40 were considered as undetectable.

### 2.5 Nanoparticle tracking analysis

Exosome concentration and size distribution extracted from patients’ plasma samples were measured using a ZetaView PMX220 TWIN instrument (Particle Metrix GmbH, Germany). Exosomes were diluted in PBS (from 1:100 to 1:1000) to achieve the optimal concentration (10^6–^10^9^ particles/ml) with each sample measured 3 times. Video data were acquired using the following settings: temperature: 22 °C; positions: 11; camera sensitivity: 68; shutter value: 100; laser wavelength: 488nm; filter wavelength: scatter. Data were analyzed using the ZetaView NTA software (version 8.05.16) with the following post-acquisition settings: minimum brightness: 30; max area: 1000; min area: 10 and classes/decade: 64.

### 2.6 Statistics

Calculated mean Ct values, melt curves and standard curve for each target were obtained from instrument software, and those Ct values were used for further analysis. Duplicate Ct values for each sample were averaged. Ct values for each miRNA were normalized to average Ct values of endogenous miRNAs (rRNA 5S). Statistical analysis was done on delta Ct (ΔCt) values. 2-way ANOVA with repeated measures test was used to establish significant differences in miRNAs levels between 3 groups at 3 different timepoints and to establish the impact of clinical factors on miRNA expression. One-way ANOVA was used to establish differences in miRNAs expression in the same cohort between different timepoints. Multiple.

t-test (Student’s t-test) were performed to establish differences in miRNAs expression between groups at 3 different timepoints. T-test was performed to establish difference in miRNA expression between two groups. A p-value of <0.05 was considered significant. Statistical analyses and data plotting were conducted using GraphPad Prism^®^ software (version 9; GraphPad software, San Diego, CA, USA). For the comparison of miRNA-194-3p between PTLD-HSCT, Non-PTLD-HSCT and healthy donors, we additionally identified and removed 2 outlier data points by the ROUT method (Q = 1%). The statistical tests were corrected for multiple comparison using hypothesis testing by Sidak (for 2-way ANOVA) or Turkey correction (One-way ANOVA).

## 3 Results

### 3.1 Participant characteristics

A total of 23 PTLD-HSCT, 25 Non-PTLD-HSCT, 10 PTLD-SOT-EBV(+), 8 PTLD-SOT-EBV(-) and 25 HD were included in the study. The mean age was comparable among PTLD-HSCT and Non-PTLD-HSCT group (52 *vs* 53 years-old). The PTLD-HSCT cohort comprised 12 males and 11 females, whereas the Non-PTLD-HSCT group included 16 males and 9 females. The average time to PTLD diagnosis after HSCT was around 6 to 7 months, however most patients (18/23 [78%]) were diagnosed already within the first 6 months post-HSCT. Moreover, PTLD-HSCT patients were checked for EBV infection by *in situ* hybridization (ISH) staining for EBV-encoded small RNA (EBER) of available biopsies. Out of 23 PTLD-HSCT patients, 18 had available biopsies for EBER ISH staining with 16 of them having a positive staining result, indicating latent EBV infection and 2 biopsy samples having a negative EBER ISH staining result. Both recipients and donors were checked for the presence of CMV prior to HSCT. Detailed clinical information including CMV status and immunosuppression strategies are summarized in **Table 1**. Similarly, among SOT recipients, the mean age between PTLD-SOT-EBV(+) and.

**Table 1:** Characteristics of hematopoietic stem cell transplant recipients.

Characteristics	PTLD-HSCT n=23	Non-PTLD-HSCT n=25
Demographics		
Age, mean, median (range)	47.8; 52 (16-67)	45.4; 53 (0-68)
Sex (n)	M (12); F (11)	M (16); F (9)
Average time		
Average time from HSCT to PTLD diagnosis in months (range)	6.7 (1-54)	
EBV *in situ* hybridization result		
Positive	16 (69.56%)	
Negative	2 (8.70%)	
No biopsy available	5 (21.74%)	25 (100%)
CMV status (n) %		
**CMV mismatch**	**6 (26.10%)**	**7 (28.00%)**
Donor-positive/Recipient-negative [D+/R-]	4 (17.40%)	1 (4.00%)
Donor-negative/Recipient-positive [D-/R+]	2 (8.70%)	6 (24.00%)
**CMV match**	**4 (17.40%)**	**15 (60.00%)**
Donor-negative/Recipient-negative [D-/R-]	3 (13.05%)	7 (28.00%)
Donor-positive/Recipient-positive [D+/R+]	1 (4.35%)	8 (32.00%)
**Not available**	**13 (56.50%)**	**3 (12.00%)**
Hematopoietic progenitor cell source (HPC) (n) %		
HPC from apheresis (HPC-A)	18 (78.26%)	22 (88.00%)
HPC from bone marrow (HPC-M)	5 (21.74%)	3 (12.00%)
HSCT subtype (n) %		
Matched unrelated donor (MUD)	16 (69.57%)	12 (48%)
Matched related donor (MRD)	4 (17.39%)	11 (44%)
Haploidentical (Haplo-id)	3 (13.04%)	2 (8%)
Umbilical cord blood (UCB)	1 (4.35%)	
Underlying disorder (n) %		
**Lymphoid malignancies (L)**	**5 (21.74%)**	**5 (20.00%)**
Hodgkin Lymphoma (HL)	1 (4.35%)	2 (8.00%)
T-cell/NK-cell Lymphoma	2 (8.70%)	1 (4.00%)
Mantle Cell Lymphoma (MCL)	1 (4.35%)	
Acute lymphoblastic leukemia (ALL)	1 (4.35%)	2 (8.00%)
**Myeloid malignancies (M)**	**12 (52,17%)**	**16 (64.00%)**
Acute Myeloid leukemia (AML)	6 (26.09%)	11 (44.00%)
Myelodysplastic syndrome (MDS)	2 (8.70%)	3 (12.00%)
Myelofibrosis	1 (4.35%)	
Chronic myelogenous leukemia (CML)	1 (4.35%)	
Multiple Myeloma (MM)		1 (4.00%)
**Others**	**2 (8.70%)**	**4 (12.00%)**
Aplastic **anaemia** (AA)	2 (8.70%)	1 (4.00%)
Cystinosis		1 (4.00%)
Inherited Disorder		2 (8.00%)
**Not available**	**6 (26.09%)**	
GvHD prophylaxis (n) %		
Use of Anti-thymocyte globulin (ATG)	6 (26.09%)	13 (52.00%)
Use of Cyclosporine A (CsA)	8 (34.78%)	20 (80.00%)
Use of methotrexate (MTX)	5 (21.74%)	3 (12.00%)
Use of mycophenolate mofetil (MMF)	4 (17.39%)	13 (52.00%)
Use of Cyclophosph**amid**e (CP)		3 (12.00%)
Use of Prograft (P)		1 (4.00%)
Not available	14 (60.87%)	4 (16.00%)
Ablative conditioning (n) %		
Yes	7 (30.34%)	9 (36.00%)
No	16 (69.66%)	16 (64.00%)
PTLD classification (n) %		
**Non-destructive**	**3 (13.04%)**	
Plasmatic hyperplasia	3 (13.04%)	
**Destructive**	**15 (65.22%)**	
**Monomorphic**	**15 (65.22%)**	
Diffuse Large B-cell Lymphoma (DLBCL)	10 (43.48%)	
High-Grade B-Cell Lymphoma (HGBCL)	1 (4.35%)	
Plasmablastic lymphoma (PBL)-PTLD	4 (17.39%)	
**No biopsy available**	**5 (21.74%)**	
Outcome at last follow up date (n) %		
Alive	11 (47.83%)	11 (44.00%)
Dead	12 (52.17%)	14 (56.00%)

Bold entries represent overarching categories, with subsequent entries showing corresponding subcategories.

PTLD-SOT-EBV(-) was comparable (50,5 *vs* 54,5 years-old). The PTLD-SOT-EBV(+) group consisted of 4 males and 4 females, while the PTLD-SOT-EBV(-) group included 5 males and 3 females. As expected, the average time between transplantation and PTLD diagnosis was significantly different between the two groups. PTLD-SOT-EBV(+) patients developed PTLD around 7 months post-transplantation, while PTLD-SOT-EBV(-) patients diagnosis occurred substantially later, around 144 months post-SOT. Additionally, in both groups, the most common transplantation type was kidney transplantation. Detailed clinical characteristic of SOT recipients is presented in **Table 2**.

**Table 2:** Characteristics of solid organ transplant recipients.

Characteristics	PTLD-SOT-EBV(+) n=10	PTLD-SOT-EBV(-) n=8
Demographics		
Age, mean, median (range)	50,5; 58 (20-70); NA (3)	54,5; 58 (37-65); NA (1)
Sex (n)	M (4); F (6)	M (5); F (3)
Average time		
Average time from Tx to PTLD diagnosis in months (range)	6.9 (0-19)	144 (51-289)
SOT type (n) %		
Kidney	4 (40%)	5 (62.5%)
Lung	2 (20%)	1 (12.5%)
Kidney-pancreas	1 (10%)	1 (12.5%)
Heart-liver-kidney	1 (10%)	
Heart		1 (12.5%)
Liver	1 (10%)	
Not available	1 (10%)	
PTLD classification (n) %		
Diffuse Large B-cell Lymphoma (DLBCL)	9 (90%)	6 (75%)
Classical Hodgkin Lymphoma-type PTLD (HL-PTLD)		1 (12.5%)
Not available	1 (10%)	1 (12.5%)

### 3.2 Characterization of exosomes

To verify exosome size and determine their concentration, we used Nanoparticle tracking analysis (NTA). Due to limited sample availability, we were able to test 7 PTLD-HSCT (each at 3 different timepoints), 7 Non-PTLD-HSCT (each at 3 different timepoints) and 7 healthy donor samples. The mean concentration of all PTLD-HSCT samples, regardless of the timepoint, was the highest (4,35 x 10^10^ particles per ml), compared to Non-PTLD-HSCT (3,12 x 10^10^ particles per ml), and healthy donors (1,40 x 10^10^ particles per ml) (**Supplementary Figure 1****).** Looking at the concentration of exosomes per timepoint, we saw the highest concentration in T0 (at the time of transplantation), slowly declining at T1 (3 months post-HSCT) and T2 (6 months post-HSCT) with 4,64 x 10^10^, 4,42 x 10^10^ and 4,03 x 10^10^, particles per ml, respectively. However, Non-PTLD-HSCT exosome concentration based on T0, T1 and T2 was the lowest at T0 (2,64 x 10^10^ particles per ml) and increasing at T1 and T2 (3,31 x 10^10^ and 3,40 x 10^10^ particles per ml, respectively) (**Supplementary Figure 1****).**

### 3.3 miRNA expression in the exosomes of post-transplant patients

**Figure 1:**
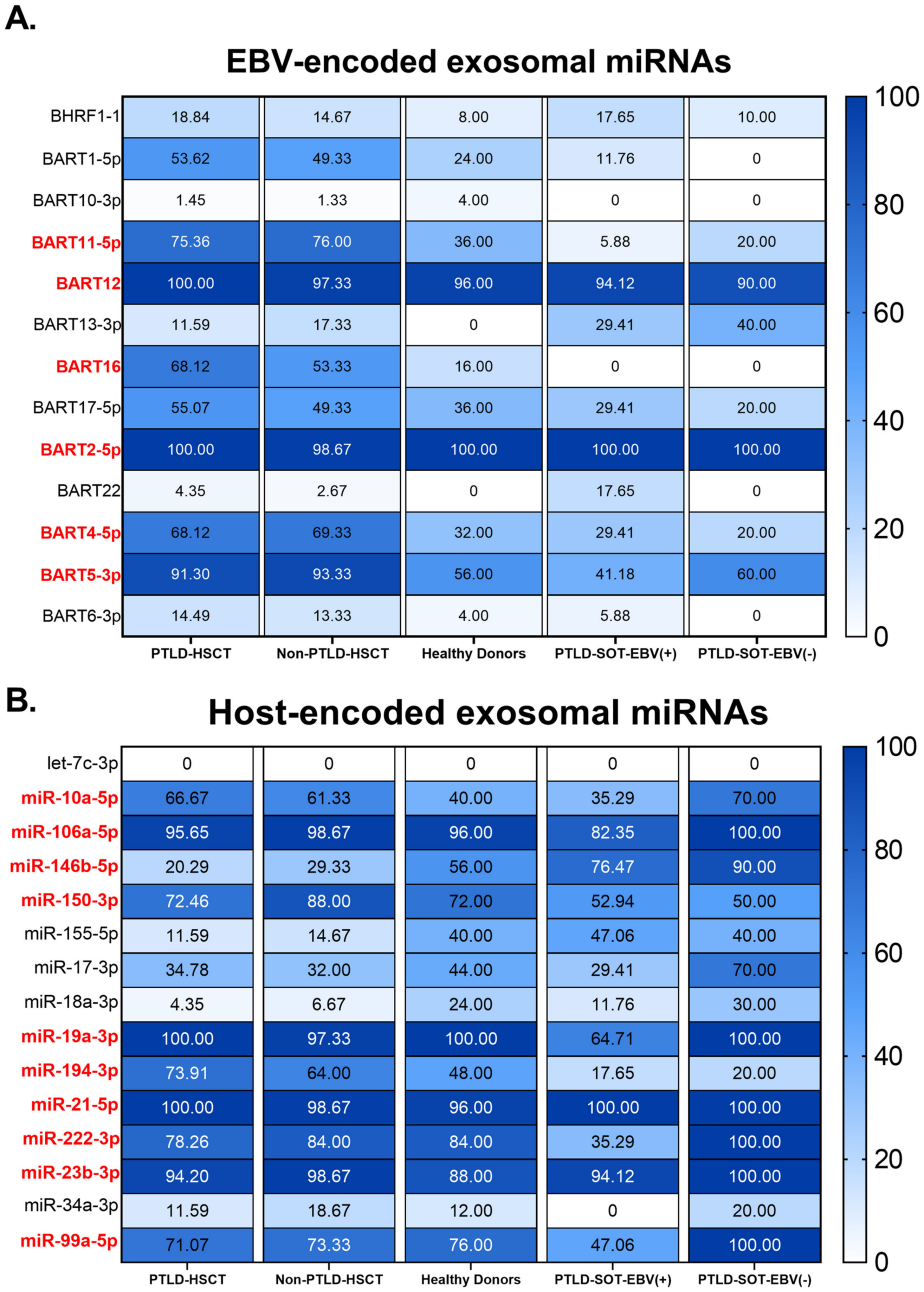
Expression profile of exosomal miRNAs. Heatmaps shows the percentage of **(A)** 13 EBV-encoded miRNA and **(B)** 15 host-encoded miRNA detection in each patients group (PTLD-HSCT, Non-PTLD-HSCT, Healthy donors, PTLD-SOT-EBV(+), PTLD-SOT-EBV(-)). The percentage of miRNA detection presented are calculated from combined timepoints in each respective research group. MiRNAs selected for further analysis are highlighted in red.

First, we quantified the expression level of 28 exosomal miRNAs (13 EBV-encoded and 15 host-derived miRNAs), selected based on prior literature. Candidate miRNAs were selected by a literature search of previously published studies investigating miRNAs expression associated with either EBV infection, EBV-associated pathologies, cancer development and/or hematological malignancies (**Supplementary Table 2**) (13–58). The exosomal miRNAs were extracted from the plasma of 23 PTLD-HSCT, 25 Non-PTLD-HSCT at 3 different timepoint, 10 PTLD-SOT-EBV(+), 8 PTLD-SOT-EBV(-) at various timepoints and from 25 healthy donors at one timepoint. miRNAs detected in more than 50% of samples in at least two patients groups were included for further investigation. Based on this criteria, 16 miRNAs were included in the final analysis, comprising 10 host-encoded (miR-10a-5p, miR-106a-5p, miR-146b-5p, miR-150-3p, miR-19a-3p, miR-194-3p, miR-21-5p, miR-222-3p, miR-23b-3p, miR-99a-5p) and 6 EBV-encoded (BART11-5p, BART12, BART16, BART2-5p, BART4-5p, BART5-3p) (**Figure 1****).**

### 3.4 Exosomal EBV-encoded miRNA expression in HSCT recipients

**Figure 2:**
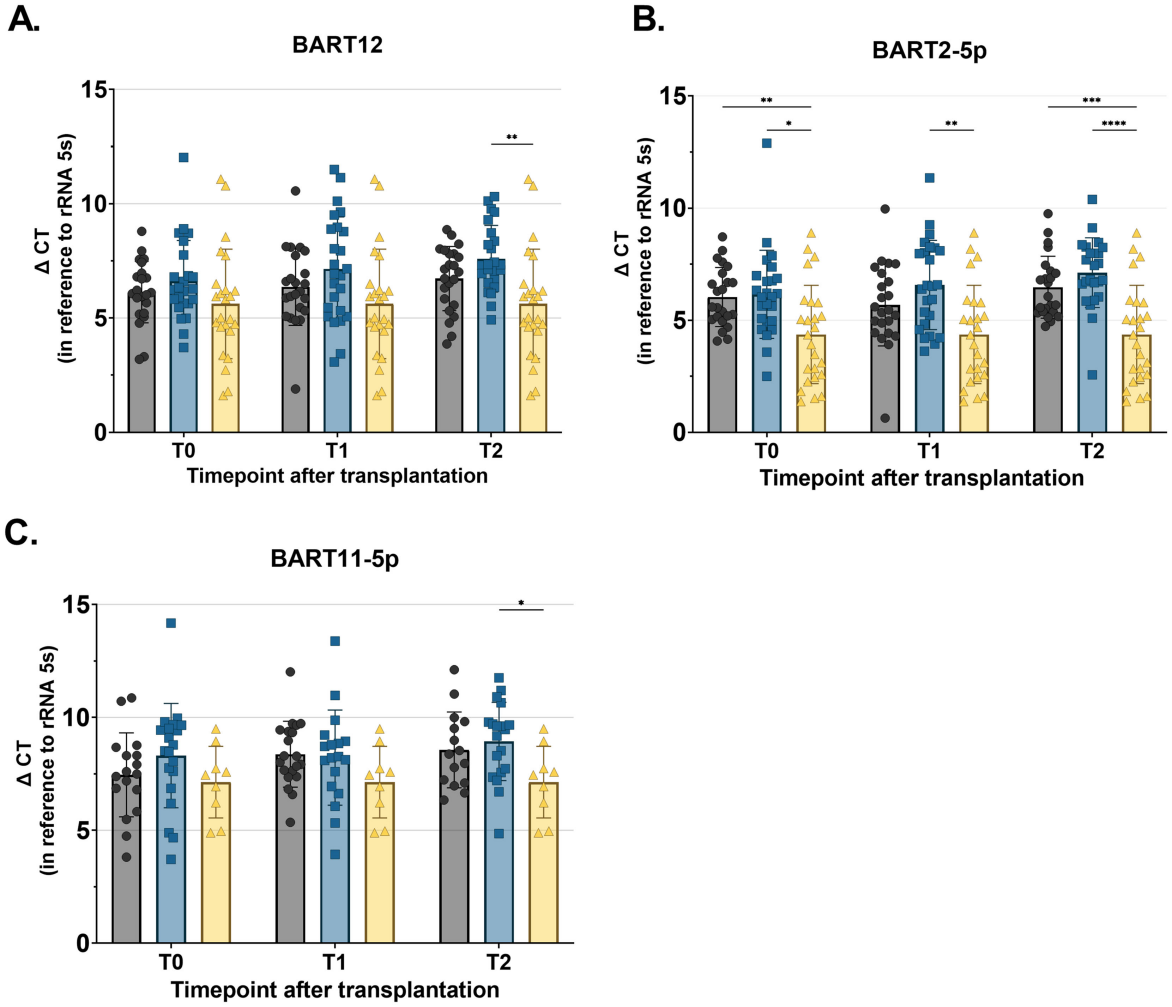
EBV-encoded miRNAs expression of post-HSCT recipients. The comparison of EBV-encoded miRNAs **(A)** BART12, **(B)** BART2-5p, **(C)** BART11-5p expression between 23 PTLD-HSCT (black), 25 Non-PTLD-HSCT (blue) and 25 Healthy donors (yellow) at 3 different timepoints (T0 [at the time of HSCT, T1 [3 months post-HSCT] and T2 [6 months post-HSCT]). Significance was determined as a P value of <0.05 and SD is shown. (*) p ≤0.05, (**) p<0.01, (***) p<0.001, (****) p<0.0001.

When comparing the expression levels of EBV-encoded miRNAs among PTLD-HSCT and Non-PTLD-HSCT across different timepoints, no significant differences were observed at any evaluated time point (**Figure 2**, **Supplementary Figure 2**). However, significant differences were detected when comparing HSCT recipients (independently of the development of PTLD) to healthy donors for 3 out of 6 of the selected EBV-encoded miRNAs. At T2, the expression levels of BART11-5p and BART12 differed significantly between the Non-PTLD-HSCT group and healthy donors. Specifically, Non-PTLD-HSCT patients exhibited higher ΔCt values, indicating lower expression levels relative to healthy donors (**Figure 2****).** In addition, significant differences in BART2-5p expression were observed between healthy donors and both PTLD-HSCT and Non-PTLD-HSCT patients at T0 and T2 and between healthy donors and Non-PTLD-HSCT group at T1. Post-HSCT patients demonstrated reduced BART2-5p expression compared to healthy donors (**Figure 2****).** Additionally, we also compared the EBV-encoded miRNA expression between 21 PTLD-HSCT patients, for whom the presence of EBV was confirmed by EBER *in situ* hybridization, with 2 PTLD-HSCT patients who had negative EBER staining results (**Supplementary Figure 3**). No significant differences in EBV-encoded miRNAs were seen between these two HSCT groups, most likely due to the small sample size of the HSCT patients with negative EBV staining for their biopsies. PTLD-HSCT patients with EBV negative staining results had comparable miRNA levels at T0 and T1; however at T2, they had higher expression of miRNAs BART11-5p, BART12, BART16, BART4-5p or lower expression of miRNAs BART2-5p and BART5-3p than PTLD-HSCT patients with EBV-positive biopsy (**Supplementary Figure 3**).

### 3.5 Exosomal host-encoded miRNA expression in HSCT recipients

**Figure 3:**
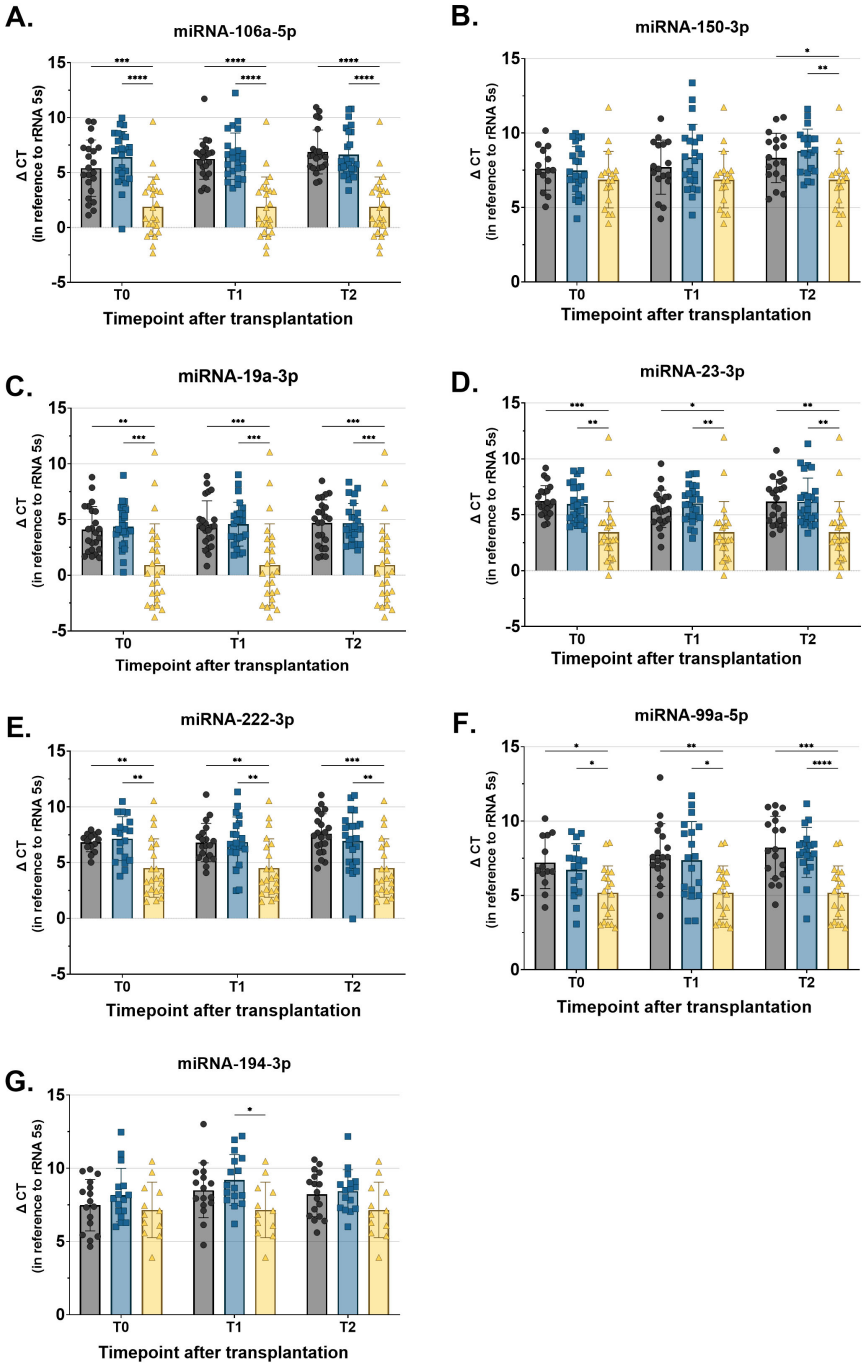
Host-encoded miRNAs expression of post-HSCT recipients. The comparison of host-encoded miRNAs **(A)** miR-106a-5p, **(B)** miR-150-3p, **(C)** miR19a-3p, **(D)** miR-23-3p, **(E)** miR-222-3p, **(F)** miR-99a-5p, **(G)** miR-194-3p expression between 23 PTLD-HSCT (black), 25 Non-PTLD-HSCT (blue) and 25 Healthy donors (yellow) at 3 different timepoints (T0 [at the time of HSCT, T1 [3 months post-HSCT] and T2 [6 months post-HSCT]). Significance was determined as a P value of <0.05 and SD is shown. (*) p ≤0.05, (**) p<0.01, (***) p<0.001, (****) p<0.0001.

Similar to EBV-encoded miRNAs, no significant differences in the expression of host-encoded miRNAs were observed between the PTLD-HSCT and Non-PTLD-HSCT groups at any evaluated time point (**Supplementary Figure 4**, **Figure 3****).** However, several (5 out of 10) host-derived miRNA (miR-106a-5p, miR-19a-3p, miR-23b-3p, miR-222-3p, and miR-99a-5) showed significantly different expression levels when comparing healthy donors with both PTLD-HSCT and Non-PTLD-HSCT patients across all time points. Healthy donors exhibited lower ΔCt values, corresponding to higher expression levels compared to post-HSCT patients (**Figure 3****).** Additionally, miR-150-3p expression at T2 differed significantly between healthy donors and both HSCT recipient groups and a significant difference in miR-194-3p expression was also observed at T1 between healthy donors and the Non-PTLD-HSCT group (**Figure 3****).** Additionally, we also compared the host-encoded miRNA expression between 21 PTLD-HSCT patients, who had confirmed EBV presence in biopsies by EBER *in situ* hybridization, with 2 PTLD-HSCT patients who had negative staining results (**Supplementary Figure 5**). Again, no significant differences between the groups was observed likely due to the small sample size. PTLD-HSCT patients with EBV negative staining results had comparable miRNA levels for miR-19a-3p, miR-194-3p, miR-222-3p, miR-10a-5p, miR-106a-5p to that of PTLD-HSCT patients with EBV-positive biopsies at all timepoints, with miR-106a-5p having a slightly lower expression at T0 in patients with EBV negative staining results. miR-150-3p and miR-23-3p, miR-21-5p, miR-99a-5p expression levels were higher at T0, lower at T1 and similar, or slightly higher at T2 in PTLD-HSCT patients with EBV negative biopsy staining compared to PTLD-HSCT patients with EBV detection by staining (**Supplementary Figure 5**).

### 3.6 Exosomal miRNA expression in SOT recipients developing PTLD relative to the time after transplantation

We next investigated differences in exosomal miRNA expression in PTLD patients following SOT. Here, we have also used the same previously selected panel of miRNAs including 10 host-encoded (miR-10a-5p, miR-106a-5p, miR-146b-5p, miR-150-3p, miR-19a-3p, miR-194-3p, miR-21-5p, miR-222-3p, miR-23b-3p, miR-99a-5p) and 6 EBV-encoded (BART11-5p, BART12, BART16, BART2-5p, BART4-5p, BART5-3p) (**Figure 1**). Because multiple longitudinal samples were available from the same patients, the samples were initially categorized according to the time of transplantation: T0 (at the time of transplantation), T1 (3 months post-SOT), T2 (6 months post-SOT), and T3 (>19 months post-SOT). Such broad timepoint range is owing to the fact that EBV(-) PTLD is often diagnosed month or years following SOT.

Due to the limited number of SOT PTLD patients and the variability in the timing of PTLD diagnosis post transplantation, the distribution of samples across the timepoints T0, T1, T2 and T3 was uneven. At T0, 6 samples were available from PTLD-SOT-EBV(+) patients and 2 from PTLD-SOT-EBV(−) patients; at T1, 7 and 1 samples, at T2, respectively; 2 samples from PTLD-SOT-EBV(+) patients only; and at T3, 2 samples from PTLD-SOT-EBV(+) and 7 from PTLD-SOT-EBV(−) patients. Given this imbalance and the small sample size within subgroups, comparisons based on time of SOT were not optimal for robust miRNA expression analysis (**Supplementary Figures 6**, **7****).** Therefore, we decided to compare the exosomal miRNA expression in SOT recipients in function of the timing of PTLD diagnosis rather than the timing of transplantation.

### 3.7 Exosomal miRNA expression in SOT recipients developing PTLD relative to the time of PTLD diagnosis

Samples from PTLD-SOT-EBV(+) and PTLD-SOT-EBV(−) patients were subsequently categorized according to their timing relative to PTLD diagnosis: before, at, and after PTLD diagnosis. In the PTLD-SOT-EBV(+) group, 8 samples were collected before diagnosis, 5 at diagnosis, and 2 after diagnosis. In the PTLD-SOT-EBV(−) group, 5 samples were obtained before diagnosis, 3 at diagnosis, and 2 after diagnosis.

**Figure 4:**
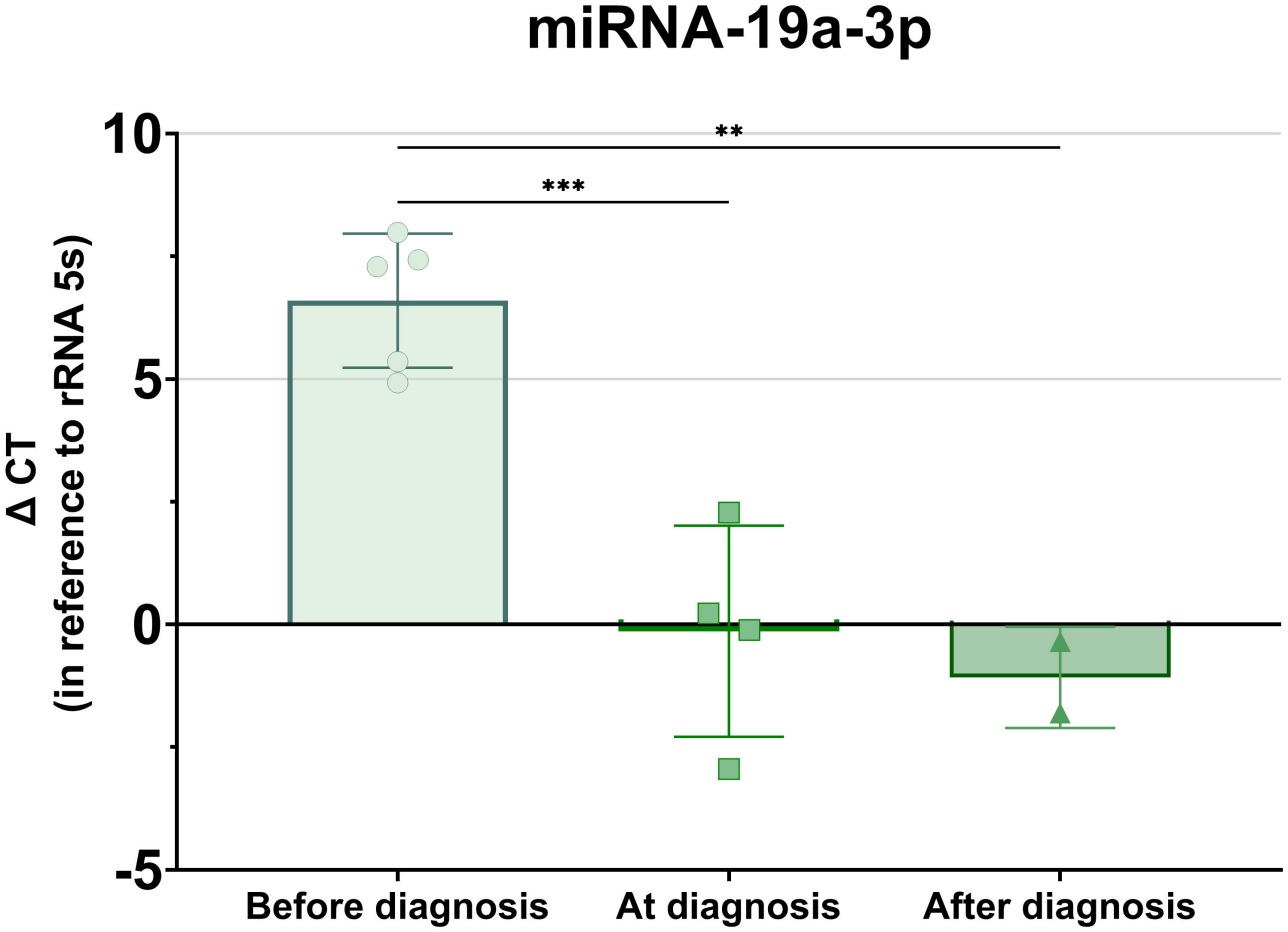
Host-encoded miRNA-19a-3p expression in PTLD-SOT-EBV(+) relative to PTLD diagnosis time. The comparison of host-encoded miR-19a-3p, expression in 10 PTLD-SOT-EBV(+) at 3 different timepoints (before, at and after diagnosis). Significance was determined as a P value of <0.05 and SD is shown. (**) p<0.01, (***) p<0.001.

**Figure 5:**
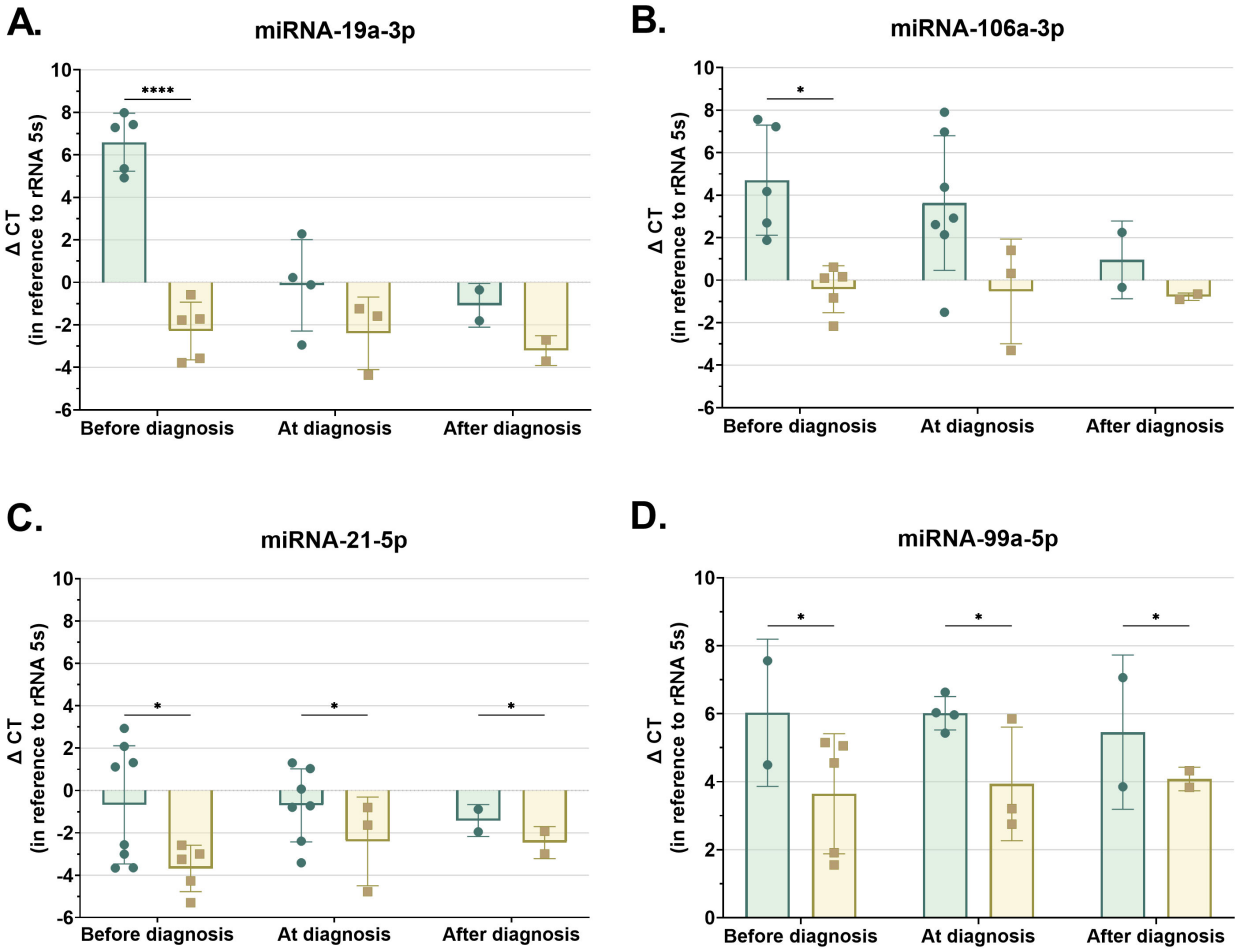
Comparison of miRNAs expression between PTLD patients after HSCT or SOT in function of PTLD diagnosis time. The comparison of host-encoded **(A)** miR-19a-3p, **(B)** miR-10a-5p, **(C)** miR-106a-5p, **(D)** miR-21-5p, **(E)** miR-23b-3p, **(F)** miR-99a-5p and EBV-encoded **(G)** BART2-5p expression in 23 PTLD-HSCT (black), 10 PTLD-SOT-EBV(+) (green) and in 8 PTLD-SOT-EBV(-) (yellow) at 3 different timepoints (before, at and after PTLD diagnosis). Significance was determined as a P value of <0.05 and SD is shown. (*) p ≤0.05, (****) p<0.0001.

We then assessed whether exosomal miRNA expression, of both host- and EBV-encoded miRNAs, was different in function of PTLD diagnosis. A significant difference was observed for miR-19a-3p expression in PTLD-SOT-EBV+ patients, with lower expression levels prior to diagnosis compared to samples collected at or after diagnosis (**Figure 4****).** No other significant differences were detected in the expression of either EBV- or host-encoded miRNAs in PTLD-SOT-EBV(+) or PTLD-SOT-EBV(−) patients when comparing samples obtained before, at, and after PTLD diagnosis (**Supplementary Figures 8**-**11****).** We further compared both host and EBV-encoded miRNAs expression between PTLD-EBV(+) and PTLD-EBV(-) SOT patients at different timepoints relative to PTLD diagnosis. Significant differences were observed for miR-19a-3p and miR-106a-5p between PTLD-SOT-EBV(+) and PTLD-SOT-EBV(-) before diagnosis, with their expression being lower in EBV(+) PTLD patients compared to EBV(-) PTLD patients (**Figure 5****).** Additionally, higher miRNA-21-5p and miR-99a-5p expression was seen in PTLD-SOT-EBV(-) patients compared to PTLD-SOT-EBV(+) patients before, at and after diagnosis (**Figure 5****).** The expression of the rest of host-encoded miRNAs and EBV-encoded miRNAs was not different between the two patients group before, at or after the diagnosis (**Supplementary Figure 12****).**

### 3.8 Comparison of miRNA expression between HSCT and SOT recipients developing PTLD relative to the time of PTLD diagnosis

**Figure 6:**
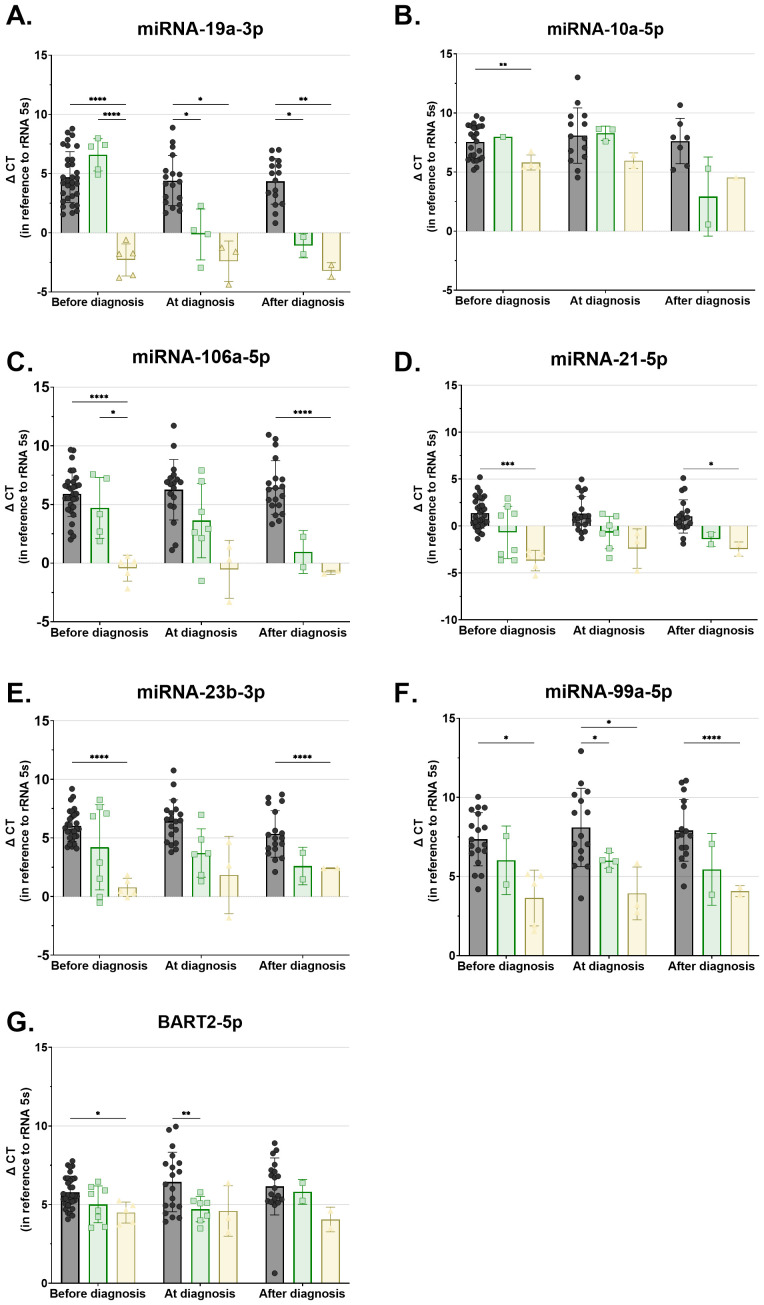
Comparison of miRNAs expression between PTLD patients after HSCT or SOT in function of PTLD diagnosis time. The comparison of host-encoded **(A)** miR-19a-3p, **(B)** miR-10a-5p, **(C)** miR-106a-5p, **(D)** miR-21-5p, **(E)** miR-23b-3p, **(F)** miR-99a-5p and EBV-encoded **(G)** BART2-5p expression in 23 PTLD-HSCT (black), 10 PTLD-SOT-EBV(+) (green) and in 8 PTLD-SOT-EBV(-) (yellow) at 3 different timepoints (before, at and after PTLD diagnosis). Significance was determined as a P value of <0.05 and SD is shown. (*) p ≤0.05, (**) p<0.01, (***) p<0.001, (****) p<0.0001.

Finally, we also compared the difference in miRNAs expression between PTLD patients after HSCT and SOT, for both EBV(-) and EBV(+) patients (**Figure 6**, **Supplementary Figure 13****).** We found a significant higher expression (lower ΔCT value) for miRNA-19a-3p in.

PTLD-SOT-EBV(-) compared to PTLD-SOT-EBV(+) and PTLD-HSCT at all the three timepoints (before, at, and after PTLD diagnosis) (**Figure 6**).

For most other tested miRNAs (miRNA-106a-5p, miRNA-21-5p, miRNA-23b-3p, miRNA-99a-5p), their expression was significantly lower in PTLD-SOT-EBV(-) patients compared to PTLD-HSCT and PTLD-SOT-EBV(+) patients before and after PTLD diagnosis. For two other miRNAs (miRNA-10a-5p and BART2-5p), a significant difference was seen when comparing PTLD-SOT-EBV(-) patients and PTLD-HSCT patients before but not after PTLD diagnosis. When analyzing the miRNA expression at the time of PTLD diagnosis, only significant differences were found for miRNA-19a-3p and miRNA-99a-5p expression when comparing PTLD-HSCT *versus* PTLD-SOT either EBV(+) or EBV(-) patients and for BART2-5p showing significant higher expression in PTLD-SOT –EBV(+) than in PTLD-HSCT patients (**Figure 6**).

### 3.9 Correlation of miRNA expression with clinical data in HSCT and SOT recipients

**Figure 7:**
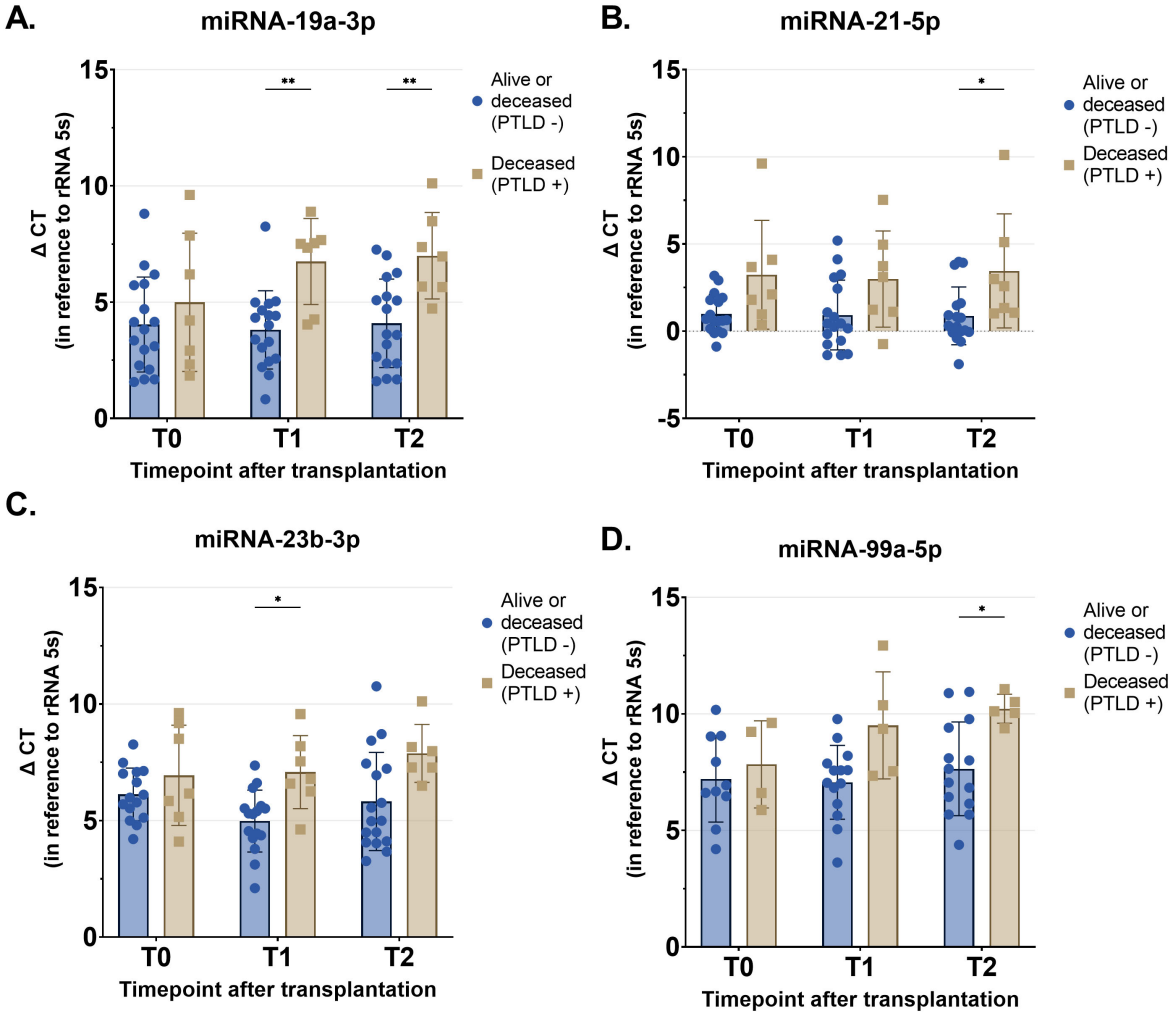
Correlation between miRNAs expression and PTLD-HSCT patients survival. The correlation between the expression of host-encoded **(A)** miR-19a-3p, **(B)** miR-21-5p, **(C)** miR-23b-3p, **(D)** miR-99a-5p in PTLD-HSCT patients and their outcome (alive or deceased without PTLD [blue] or deceased with PTLD [brown]) at T0,T1 and T2. Significance was determined as a P value of <0.05 and SD is shown. (*) p ≤0.05, (**) p<0.01.

Next, we analyzed the correlation of exosomal miRNAs expression and clinical data from HSCT recipients and PTLD patients, including PTLD classification (destructive and non-destructive PTLD), PTLD subtype (plasmatic hyperplasia, diffuse large B-cell lymphoma [DLBCL], plasmablastic lymphoma [PBL-PTLD]), HSCT subtype (matched related donor [MRD], matched unrelated donor [MUD], umbilical cord blood [UCB] and haplo-identical [haplo-id] donor), patients’ gender, ablative conditioning, GvHD (graft versus host disease) prophylaxis and outcome. Among PTLD-HSCT patients, we found a significant difference in miRNA-21-5p expression between patients that received stem cells from UCB compared to those receiving stem cells from haplo-identical donors at 3 and 6 months post transplantation (**Supplementary Figure 14****).** Moreover, Non-PTLD-HSCT patients, with haplo-identical donor had higher expression of miRNA-150-3p and of miRNA-99a-5p compared to MUD HSCT recipients at 3 months post transplantation. The expression of BART2-5p in MRD Non-PTLD-HSCT patients was higher at T0 than in those with MUD transplantation. We then investigated whether miRNA expression can be influenced by ablative conditioning prior to HSCT and by GvHD prophylaxis in the post-HSCT recipients cohort. While there was no difference between miRNA expression and the diverse GvHD prophylaxis strategies (not shown), there were significant differences in miRNA expression (**Supplementary Figure 15****).** EBV-encoded miRNAs BART16, BART2-5p, BART4-5p and host-encoded miR-106a-5p, miR-150-3p and miR-21-5p all showed lower expression in post-HSCT patients not receiving ablative conditioning prior to HSCT compared to patients who received ablative conditioning (**Supplementary Figure 15**). We have also correlated exosomal miRNAs expression with patients outcome: either alive or deceased without PTLD at the last follow-up or deceased with PTLD at the last follow-up date. Deceased patients with PTLD had lower expression of miRNA-19a-3p, miRNA-21-5p, miRNA-23b-3p and miRNA-99a-5p compared to alive or deceased patients without PTLD at T1 and T2, T2, T1 and T2, respectively (**Figure 7****).** We have also compared those miRNAs expression between alive patients (without PTLD) and deceased patients (with and without PTLD), with no significant difference between the groups. Additionally, due to low number of PTLD patients after SOT, the correlation between miRNAs expression and their clinical data such as the type of transplanted organ, could not be statistically compared.

## 4 Discussion

Exosomes are extracellular vesicles released from cells into body fluids and can transport proteins, lipids, and miRNAs (59). Their cargo is protected by the exosomal membrane, which ensures the exosomal miRNA expression stability (59). Therefore, exosomal miRNAs are good candidates for non-invasive biomarkers (59). miRNAs are small, non-coding RNAs, involved in a wide range of biological functions and are also implicated in various disorders (9). Their exact role in promoting or inhibiting oncogenesis remains unclear, with many conflicting statements in the literature (9). Moreover, the data on miRNA expression profiles in PTLD patients, especially exosome derived miRNAs, is very limited. Here, we have studied the expression of 10 host-encoded and 6 EBV-encoded miRNAs in post-HSCT or post-SOT patients, who developed PTLD.

EBV-encoded miRNAs play a critical role in immune escape, tumor development and apoptosis (60). Among them, BART12 is highly expressed in NPC and has been reported to promote cell invasion and migration in EBV-associated epithelial malignancies (60). However, in another study, BART12 was seen to inhibit cell proliferation and migration in GC (50). Additionally, BART2-5p, which can inhibit tumor suppressor genes and activate PI3K-AKT pathway, promoting PTLD progression, was found to be significantly higher expressed in PTLD patients compared to patients with chronic high EBV viral load (44). Moreover, BART2-5p plays a crucial role in immune evasion by preventing Natural Killer (NK) cells from recognizing EBV-infected cells (61). Elevated levels of BART2-5p have also been reported in EBV-associated epithelial malignancies, where it contributes to NPC and GC development (44, 62–64).

Host-encoded miRNAs act as key mediators in establishment and progression of various human cancers with its profiling being increasingly used for cancer detection, progression and treatment response (65). For instance, miRNA 106a-5p can control tumor progression and was associated with promoting tumorigenesis in gastric cancer (9, 66). Additionally, exosomal miRNA 106a-5p was found to promote NPC cell proliferation (66, 67). Moreover, miRNA-150 can induce EBV-positive BL cell differentiation (68). The upregulation of miRNA-19a was found to have a prognostic significance for onset of GvHD and to promote cell proliferation and invasion (9, 69). Furthermore, miRNA-222-3p behaves as an oncogene by promoting cancer and by increasing cell proliferation in multiple myeloma (70, 71). Lastly, high miR-99a expression was seen in AML patients after HSCT with poor outcomes (9, 72). In our cohort, we observed that miRNA-19a-3p, -21-5p, -23b-3b and -99a-5p were upregulated in deceased patients with PTLD after HSCT compared to alive or deceased patients without PTLD after HSCT, suggesting that these miRNAs may be specific to PTLD in post-transplant setting. Moreover, we observed that both BART11-5p, BART12 and BART2-5p were expressed at lower levels in PTLD patients or non-PTLD patients after HSCT compared to healthy donors but were not significantly different between PTLD and non-PTLD patients. Similarly, host-encoded miRNAs expression (miRNA-106a-5p, -150-3p, 19a-3p, 23b-3p, 222-3p, 194-3p, 99a-5p) were lower in post-HSCT patients compared to healthy donors, with no difference between PTLD and non-PTLD patients. This reduced expression may reflect the effects of intensive immunosuppressive therapy. Taken together, these findings indicate that the evaluated EBV-encoded and host-derived miRNAs are unlikely to serve as reliable biomarkers for PTLD development following HSCT. Additionally, it is known that miRNA levels can be altered during immunosuppressive treatment (73, 74). Moreover, different therapies can alter the rate of exosome production (73, 74). While we saw no differences in miRNA expression when comparing different GvHD prophylaxis, we saw significant differences in miRNA levels when investigating the effects of ablative conditioning in the HSCT cohort, with patients receiving ablative therapy, having higher miRNA expression. This could be due to myeloablative conditioning causing higher inflammation, which is reflected by the higher miRNA levels. When comparing the miRNAs expression levels between PTLD patients after SOT, we found that EBV(+) patients had lower expression of miRNA-19a-3p, -106a-3p, -21-5p and -99a-5p compared to EBV(-) PTLD patients. Similarly, Sen et al. also described a reduction in expression of miRNA-19 and -106a in EBV(+) PTLD pediatric patients after SOT compared to non-PTLD EBV(-) SOT recipients (1). These data suggest that these host-encoded miRNAs can be affected by EBV infection; however, whether these changes result from direct viral effect or indirect host response to the viral infection is not known. However, in a diffuse large B-cell lymphoma (DLBCL) study, the expression of miRNA-106a was downregulated in EBV(+) cases, compared to EBV(-) DLBCL cases (75). Thus, further studies on how EBV infection affects miRNAs expression in EBV-associated malignancies such as PTLD are crucial. Interestingly, we found that miRNA-19a-3p expression in PTLD-SOT-EBV(+) patients was significantly under-expressed before PTLD diagnosis compared to its expression levels at or after PTLD diagnosis, which can be a potential biomarker for PTLD development in EBV(+) patients after SOT. However, further and larger studies are needed to confirm this finding. Although our analysis focused on miRNAs detected in more than 50% of the samples, we acknowledge that several low-frequency miRNAs could be of potential importance.

EBV-encoded miRNAs (BART1-5p, BART22, and BART13-3p) showed interesting detection patterns across the patient cohorts, including differences between EBV(+) and EBV(-) SOT recipients and absence of detection in healthy controls. However, due to the low number of samples in which theses miRNAs were detectable and the small size of the cohorts, the data are not statistically significant and. Therefore, no conclusions regarding their value as biomarkers can be made based on the data here reported. Nevertheless, these findings suggest that low-abundant miRNAs may warrant further investigation.

Exosomes and their cargo can be easily extracted from body fluid, such as plasma or urine, without invasive procedures (76). Their cargo is protected from degradation and deterioration due to an exosomal membrane (76). Moreover, exosomes remain quite stable in stored patients samples (76). For those reasons, they are considered as attractive biomarker candidates. However, the use of exosomes as potential biomarkers has also disadvantages as their clinical utility can be limited due to lack of standardization of isolation techniques (76). Exosomal miRNAs seem to be a great biomarker candidate due to their availability by non-invasive extraction, stability in biological samples and many detection methods (77). However, against all advantages, there is still no miRNA that has been approved by the Food and Drug Administration (FDA) as a biomarker (77). This can be due to lack of standardization when it comes to both extraction and normalization methods. Another drawback is the lack of specificity of miRNAs as biomarker for a specific disease (77). Most often miRNA expression is compared between healthy donors and individuals with a certain pathology, where the difference in miRNA expression would not reflect specificity for a particular disease, but an overall pathological response (77). miRNA-21 or miR-155 are found in various disease, lacking specificity (77–79). In our study, we included PTLD patients, healthy donors as well as patient after HSCT without PTLD and saw no difference in miRNA expression between PTLD and non-PTLD patients but a significant difference in the expression with healthy donors, suggesting that those miRNAs are not specific to PTLD but rather reflect a pathological response. Besides miRNAs, exosomes released from EBV-infected cells can also transport viral proteins such as latent membrane proteins (LMPs), EBV-associated nuclear antigens (EBNAs) and EBV-encoded small RNA (EBER) (6). LMP1, a major EBV oncoprotein, crucial for B-cell transformation, is found on the membrane of exosomes (80). LMP1 positive exosomes were found to suppress T-cells and affect cell proliferation, promoting EBV pathogenesis (81–83). Moreover, EBV latently infected cells continuously produce EBERs and EBER1 positive exosomes have been found to promote tumorigenesis (6, 84). Thus, in addition to exosomal miRNAs, there are a few available biomarker candidates from the exosome cargo that could serve as specific biomarkers for EBV-associated disorders diagnosis, prognosis or response to treatment. Early detection and development of new therapy strategies targeting EBV can lead to reduced global burden of EBV-associated diseases (5). EBV infection can reshape the miRNA profile with over 60 host miRNAs being impacted (5). Despite this, the data on exosomal miRNA expression, especially in PTLD patients is greatly limited. To date, no studies have comprehensively analyzed exosomal miRNA expression in HSCT recipients with PTLD, or compared EBV-positive and EBV-negative SOT recipients with PTLD. Future research should focus on detailed characterization of additional components of the exosomal cargo to identify reliable and non-invasive biomarkers that are critical and specific for PTLD development.

## Data Availability

The original contributions presented in the study are included in the article/**Supplementary Material**. Further inquiries can be directed to the corresponding authors.
