# Supplementary material for: Exosomal miRNA expression in transplant recipients with EBV-associated post-transplant lymphoproliferative disorder
Source: Front Immunol. 2026 Jul 10;17:1862392. doi: 10.3389/fimmu.2026.1862392 (PMC13396207; doi:10.3389/fimmu.2026.1862392)
Supplement: Supplementary file 1 [file DataSheet1.docx]

**Supplementary material:**

**Supplemental Table 1. List of miRNA qPCR plate components**

| # | miRNA | Type of miRNA | Targeted Sequence | Catalogue number |
| --- | --- | --- | --- | --- |
| 1 | ebv-miR-BHRF1-1 | EBV-encoded miRNAs | UAACCUGAUCAGCCCCGGAGUU | YP00205815 |
| 2 | ebv-miR-BART1-5p |  | UCUUAGUGGAAGUGACGUGCUGUG | YP00205734 |
| 3 | ebv-miR-BART10-3p |  | UACAUAACCAUGGAGUUGGCUGU | YP02107103 |
| 4 | ebv-miR-BART11-5p |  | UCAGACAGUUUGGUGCGCUAGUUG | YP00205853 |
| 5 | ebv-miR-BART12 |  | UCCUGUGGUGUUUGGUGUGGUU | YP00205774 |
| 6 | ebv-miR-BART13-3p |  | UGUAACUUGCCAGGGACGGCUGA | YP00205795 |
| 7 | ebv-miR-BART16 |  | UUAGAUAGAGUGGGUGUGUGCUCU | YP00205838 |
| 8 | ebv-miR-BART17-3p |  | UGUAUGCCUGGUGUCCCCUUAGU | YP00205747 |
| 9 | ebv-miR-BART2-5p |  | UAUUUUCUGCAUUCGCCCUUGC | YP00205809 |
| 10 | ebv-miR-BART22 |  | UUACAAAGUCAUGGUCUAGUAGU | YP00205825 |
| 11 | ebv-miR-BART4-5p |  | GACCUGAUGCUGCUGGUGUGCU | YP00205790 |
| 12 | ebv-miR-BART5-3p |  | GUGGGCCGCUGUUCACCU | YP00205798 |
| 13 | ebv-miR-BART6-3p |  | CGGGGAUCGGACUAGCCUUAGA | YP00205777 |
| 14 | hsa-let-7c-3p | Human miRNAs | CUGUACAACCUUCUAGCUUUCC | YP00205202 |
| 15 | hsa-miR-10a-5p |  | UACCCUGUAGAUCCGAAUUUGUG | YP00204778 |
| 16 | hsa-miR-106a-5p |  | AAAAGUGCUUACAGUGCAGGUAG | YP00204563 |
| 17 | hsa-miR-146b-5p |  | UGAGAACUGAAUUCCAUAGGCUG | YP02119310 |
| 18 | hsa-miR-150-3p |  | CUGGUACAGGCCUGGGGGACAG | YP00206039 |
| 19 | hsa-miR-155-5p |  | UUAAUGCUAAUCGUGAUAGGGGUU | YP02119311 |
| 20 | hsa-miR-17-3p |  | ACUGCAGUGAAGGCACUUGUAG | YP00206008 |
| 21 | hsa-miR-18a-3p |  | ACUGCCCUAAGUGCUCCUUCUGG | YP00204523 |
| 22 | hsa-miR-19a-3p |  | UGUGCAAAUCUAUGCAAAACUGA | YP00205862 |
| 23 | hsa-miR-194-3p |  | CCAGUGGGGCUGCUGUUAUCUG | YP00204204 |
| 24 | hsa-miR-21-5p |  | UAGCUUAUCAGACUGAUGUUGA | YP00204230 |
| 25 | hsa-miR-222-3p |  | AGCUACAUCUGGCUACUGGGU | YP00204551 |
| 26 | hsa-miR-23b-3p |  | AUCACAUUGCCAGGGAUUACCAC | YP02119314 |
| 27 | hsa-miR-34a-3p |  | CAAUCAGCAAGUAUACUGCCCU | YP00206061 |
| 28 | hsa-miR-99a-5p |  | AACCCGUAGAUCCGAUCUUGUG | YP00204521 |
| 29 | UniSp6 | Template control | CUAGUCCGAUCUAAGUCUUCGA | YP00203954 |
| 30 | rRNA 5S | Reference miRNA | [GUCUACGGCCAUACCACCCUGAACGCGCCCGAUCUCGUCUGAUCUCGGAAGCUAAGCAGGGUCGGGCCUGGUUAGUACUUGGAUGGGAGACCGCCUGGGAAUACCGGGUGCUGUAGGCUUU] | YP00203906 |
| 31 | UniSp3 | Template control | CATCGATTGTACTAGGCTACGTTTTTTTTT | YP02119288 |
| 32 | Blank Spot | Negative control |  | YP02119291 |

**Supplemental Table 2. List of examined miRNAs and the article source.**

| # | miRNA | Type of miRNA | miRNA role | Source article(s) (PMID) |
| --- | --- | --- | --- | --- |
| 1 | ebv-miR-BHRF1-1 | EBV-encoded miRNAs | Activation of PI3K-AKT pathway, contributing to the development of PTLD. Associated with p53 aberrations in CLL. | 37419372  31671936 |
| 2 | ebv-miR-BART1-5p |  | Promotes angiogenesis and tumor metastasis in NPC. | 30557400  26701721 |
| 3 | ebv-miR-BART10-3p |  | Promotes metastasis of EBV-associated GC and proliferation of NPC. | 32533512  30745852  34424090 |
| 4 | ebv-miR-BART11-5p |  | Expressed in a range of EBV-associated B-cell lymphoma. Upregulate the expression of PD-L1 in EBV-associated NPC and GC. | 23997984  36846758 |
| 5 | ebv-miR-BART12 |  | Upregulated in NPC and associated with poor prognosis. Inhibits cell proliferation and migration. | 33094864  33646408 |
| 6 | ebv-miR-BART13-3p |  | Found in circulation in EBV-associated NPC. | 30411781  32155300 |
| 7 | ebv-miR-BART16 |  | Interferes with the type I IFN signaling pathway. Induces apoptotic resistance by targeting LMP1 in NPC. | 28416598  30123354 |
| 8 | ebv-miR-BART17-3p |  | Promotes immune escape through the enhancer-mediated transcription of PD-L. | 35165282 |
| 9 | ebv-miR-BART2-5p |  | Activation of PI3K-AKT pathway, contributing to the development of PTLD. Increased in the serum of patients with preclinical NPC and the copy number positively correlates with disease progression. | 37419372  32060148 |
| 10 | ebv-miR-BART22 |  | Promotes metastasis of EBV-associated GC. Drives malignant transformation of NPC. | 32533512  35907914 |
| 11 | ebv-miR-BART4-5p |  | Downregulation inhibits proliferation and aggressiveness while promoting radiosensitivity of NPC. Regulates cell proliferation, apoptosis, and migration in GC. | 30248542  35083633 |
| 12 | ebv-miR-BART5-3p |  | Inhibits p53 expression. | 30209170 |
| 13 | ebv-miR-BART6-3p |  | Inhibits cancer cell proliferation. Affects regulation of cell growth and immune response in BL. | 32306460  28877527  24731550 |
| 14 | hsa-let-7c-3p | Human miRNAs | Repressed by BART2-5p, downregulated during EBV infection. | 37495108 |
| 15 | hsa-miR-10a-5p |  | Involved in cancer progression including gastric cancer and AML. | 39796267 |
| 16 | hsa-miR-106a-5p |  | Enhanced the proliferative ability of NPC cells. Contributes to cisplatin resistance and tumorigenesis in NPC. | 34469038  33804927 |
| 17 | hsa-miR-146b-5p |  | Upregulated in both CD4+ T-cells derived EVs and circulating EVs obtained from antiretroviral therapy-naive HIV-1-infected patients. Showed high specificity for identifying EBV+ DLBCL. | 31311940  25544772 |
| 18 | hsa-miR-150-3p |  | Re-expression induces EBV-positive BL differentiation by modulating c-Myb. | 23521217 |
| 19 | hsa-miR-155-5p |  | Expression is required for the growth of EBV-infected B cells. Significantly elevated in EBV+ B cell lymphoma cell lines and associated exosomes. | 30021904  26634702  27110708  32038504 |
| 20 | hsa-miR-17-3p |  | Suppresses the pro-inflammatory responses via NF-κB signaling. | 33623121 |
| 21 | hsa-miR-18a-3p |  | Reactivates EBV through defective DNA damage response and promotes genomic instability in EBV-associated lymphomas. | 30594162 |
| 22 | hsa-miR-19a-3p |  | Downregulated in EBV-positive NK/T-cell lymphoma compared to EBV-negative ones. | 22870299 |
| 23 | hsa-miR-194-3p |  | Attenuates IL-10 production and increases apoptosis of EBV-positive B cell lymphoma lines. | 26147452 |
| 24 | hsa-miR-21-5p |  | Upregulated in LCLs compared to B cells and in AML and AML cell lines. | 33376550  22614176 |
| 25 | hsa-miR-222-3p |  | Predicted poor survival outcomes in patients with extranodal NK/T‑cell lymphoma. Dysregulation is closely associated with various types of human cancer. | 33760107 |
| 26 | hsa-miR-23b-3p |  | Anti-apoptotic factor in gastric cancer cells by directly targeting Programmed cell death protein 4 (PDCD4), an apoptosis regulatory protein. Upregulated in DLBCL. | 29962353  21062812 |
| 27 | hsa-miR-34a-3p |  | Expression is transcriptionally controlled by p53, a crucial tumor suppressor pathway, often disrupted in cancer. | 29022903 |
| 28 | hsa-miR-99a-5p |  | Regulates proliferation, migration and invasion abilities of human carcinoma cells. | 28592118 |

**
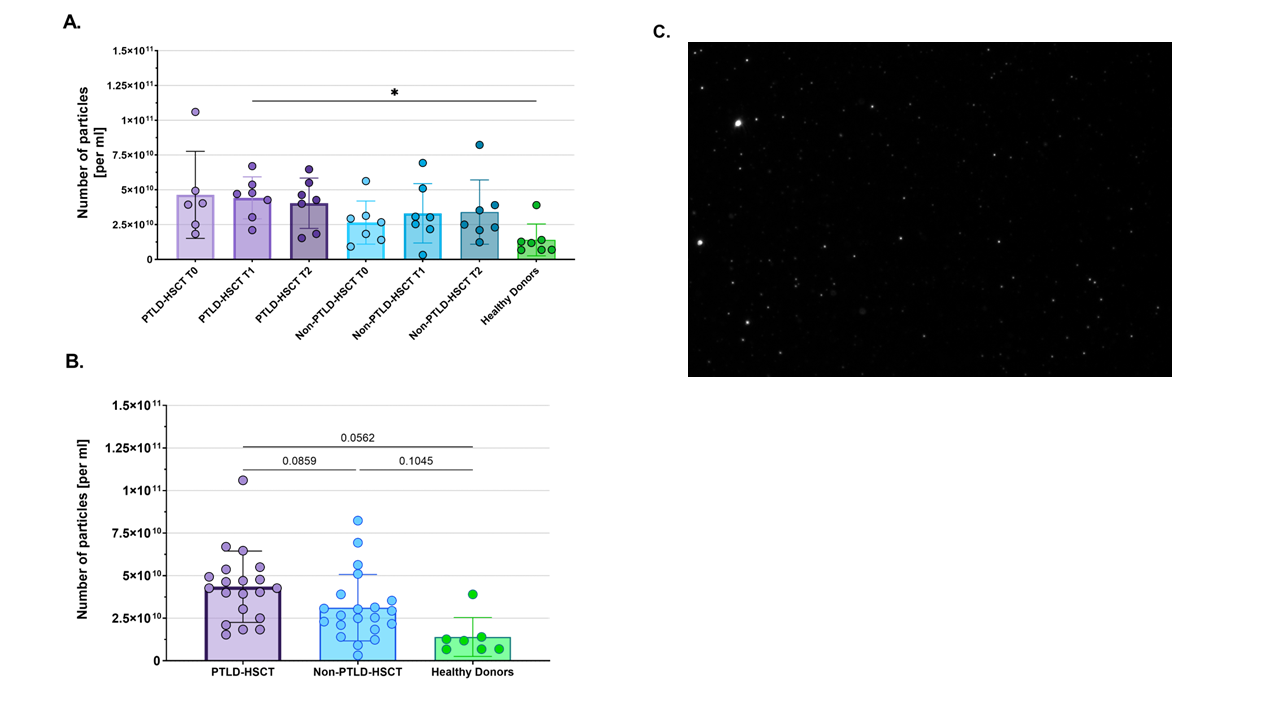
**

**Supplementary Figure 1. Exosome characterization by nanoparticle tracking analysis.** Exosome concertation of **(A) (B)** 7 PTLD-HSCT8 (purple), 7 Non-PTLD-HSCT (blue) and 7 healthy donors (green) at **(A)** at 3 different timepoints (T0 [at the time of HSCT, T1 [3 months post-HSCT] and T2 [6 months post-HSCT]) and **(B)** their overall concentration. **(C)** Representative picture of NTA**.** Significance was determined as a P value of <0.05 and SD is shown. (*) p ≤0.05, (**) p<0.01, (***) p<0.001, (****) p<0.0001.


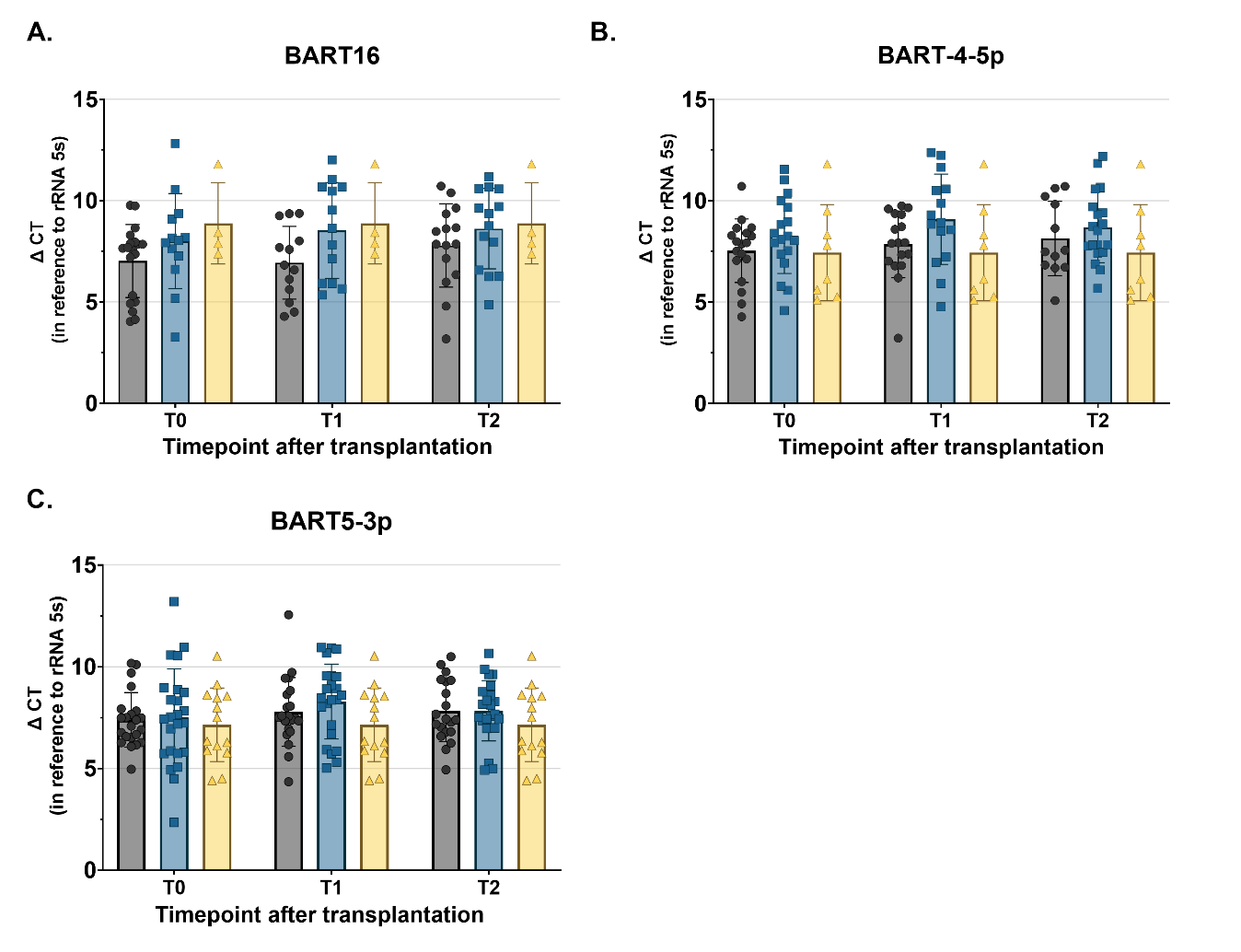
**Supplementary Figure 2. EBV-encoded miRNAs expression in HSCT recipients.** The comparison of EBV-encoded miRNAs **(A)** BART16, **(B)** BART4-5p, **(C)** BART5-3p expression between 23 PTLD-HSCT (black), 25 Non-PTLD-HSCT (blue) and 25 Healthy donors (yellow) at 3 different timepoints (T0 [at the time of HSCT, T1 [3 months post-HSCT] and T2 [6 months post-HSCT]).


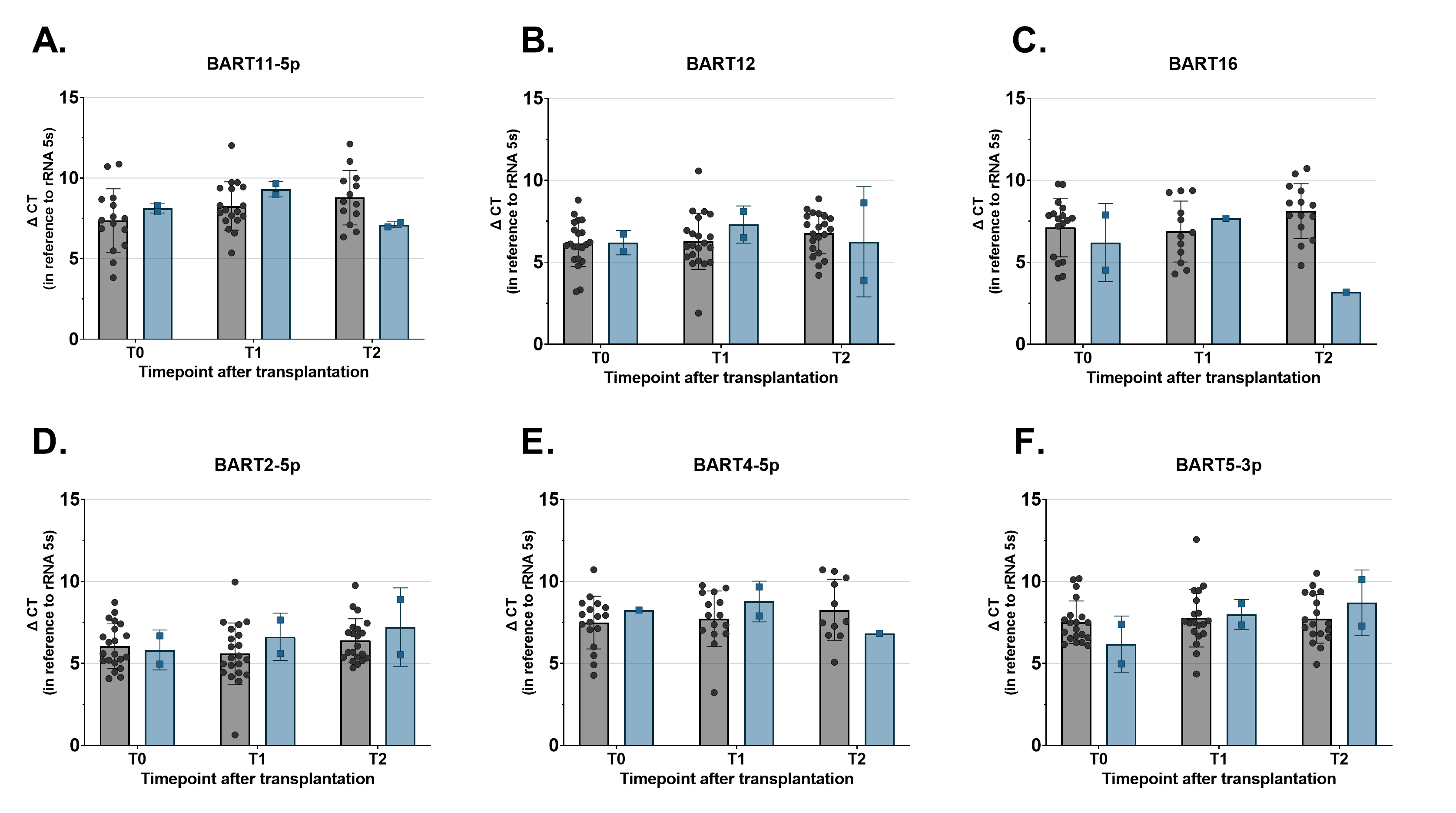


**Supplementary Figure 3. EBV-encoded miRNAs expression in PTLD-HSCT patients depending on their biopsy staining result.** The comparison of EBV-encoded miRNAs **(A)** BART11-5p, **(B)** BART12, **(C)** BART16, **(D)** BART2-5p, **(E)** BART4-5p, **(F)** BART5-3p expression between 21 PTLD-HSCT patients with a positive EBV staining of the biopsy (black) and 2 PTLD-HSCT patients with a negative EBV staining of the biopsy (blue) at 3 different timepoints (T0 [at the time of HSCT, T1 [3 months post-HSCT] and T2 [6 months post-HSCT]).


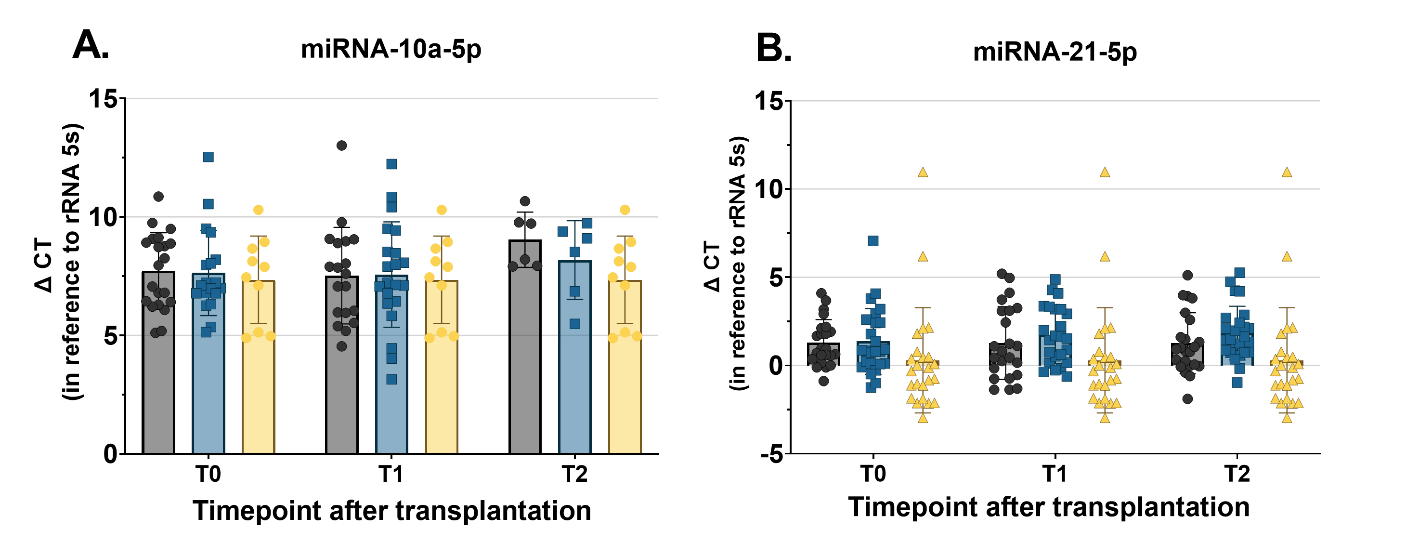


**Supplementary Figure 4. Host-encoded miRNAs expression in HSCT recipients.** The comparison of host-encoded miRNAs **(A)** miR-10a-5p, **(B)** miR-21-5p expression between 23 PTLD-HSCT (black), 25 Non-PTLD-HSCT (blue) and 25 Healthy donors (yellow) at 3 different timepoints (T0 [at the time of HSCT, T1 [3 months post-HSCT] and T2 [6 months post-HSCT]).


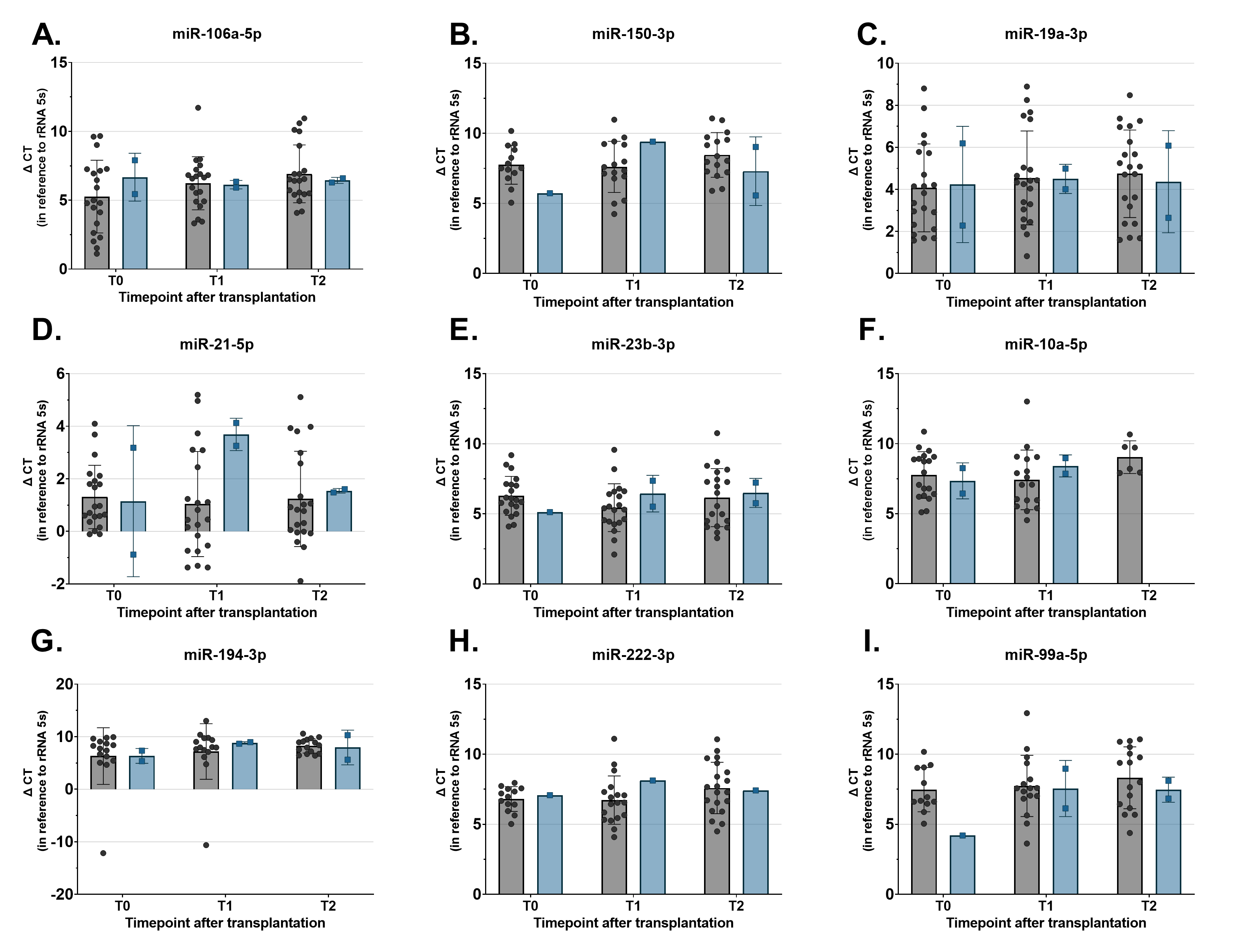


**Supplementary Figure 5. Host-encoded miRNAs expression in PTLD-HSCT patients depending on their biopsy staining result.** The comparison of host-encoded miRNAs **(A)** miR-106a-5p, **(B)** miR-150-3p, **(C)** miR-19a-3p, **(D)** miR-21-5p, **(E)** miR-23b-3p, **(F)** miR-10a-5p, **(G)** miR-194-3p, **(H)** miR-222-3p, **(I)** miR-99a-5p expression between 21 PTLD-HSCT patients with a positive EBV staining of the biopsy (black) and 2 PTLD-HSCT patients with a negative EBV staining of the biopsy (blue) at 3 different timepoints (T0 [at the time of HSCT, T1 [3 months post-HSCT] and T2 [6 months post-HSCT]).


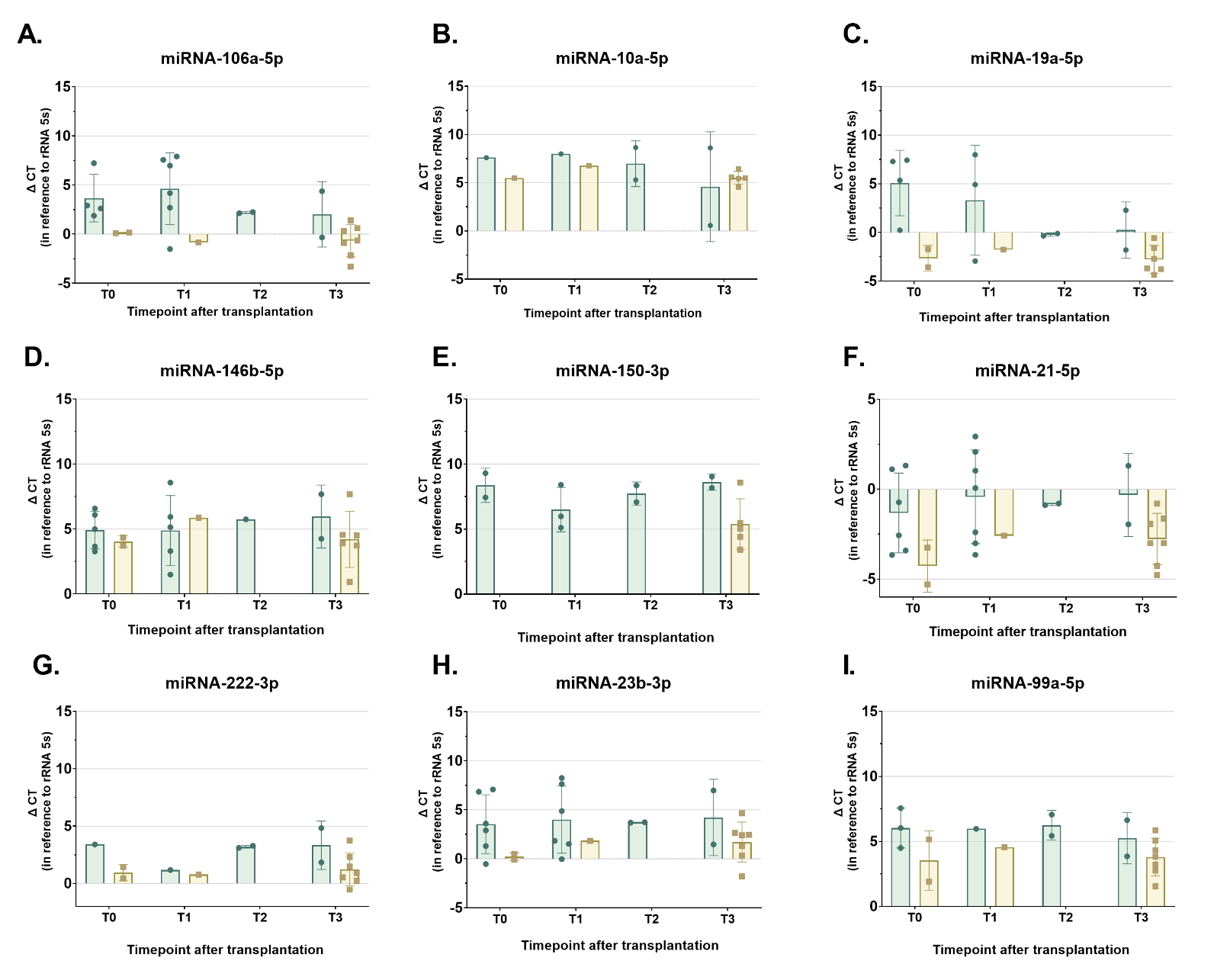
**Supplementary Figure 6. Host-encoded miRNAs expression in SOT recipients at transplantation time (T0) and at 3 (T1), 6 (T2), >19 (T3) months post-transplantation.** The comparison of host-encoded miRNAs **(A)** miR-106a-5p, **(B)** miR-10a-5p, **(C)** miR-19a-5p, **(D)** miR-146b-5p, **(E)** miR-150-3p, **(F)** miR-21-5p, **(G)** miR-222-3p, **(H)** miR-23b-3b **(I)** miR-99a-5p expression between 10 PTLD-SOT-EBV(+) (green) and 8 PTLD-SOT-EBV(-) (yellow) at 4 different timepoints (T0 [at the time of SOT, T1 [3 months post-SOT], T2 [6 months post-SOT] and T3 [>19 months post-SOT]).


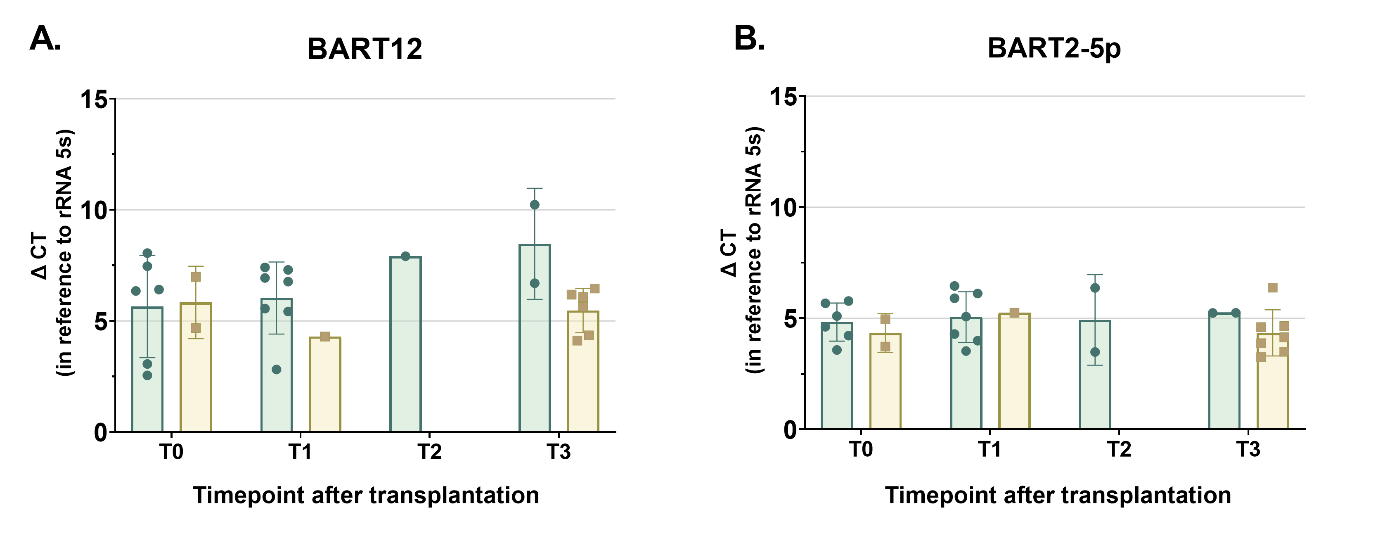
**Supplementary Figure 7. EBV-encoded miRNAs expression in SOT recipients at transplantation (T0) and at 3 (T1), 6 (T2), >19 (T3) months post-transplantation.** The comparison of EBV-encoded miRNAs **(A)** BART12, **(B)** BART2-5p, expression between 10 PTLD-SOT-EBV(+) (green) and 8 PTLD-SOT-EBV(-) (yellow) at 4 different timepoints (T0 [at the time of SOT, T1 [3 months post-SOT], T2 [6 months post-SOT] and T3 [>19 months post-SOT]).


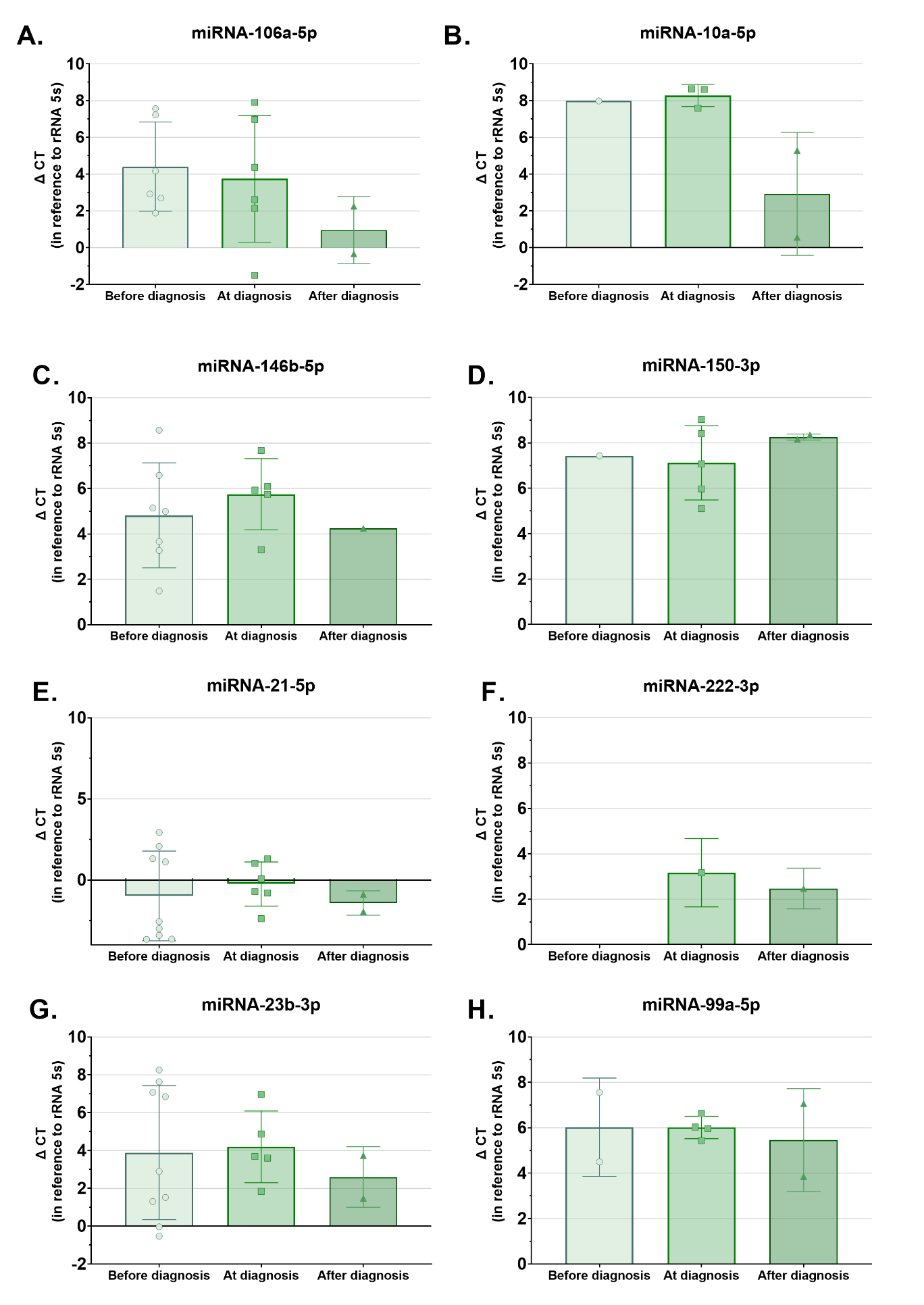
**Supplementary Figure 8. Host-encoded miRNAs expression in PTLD-SOT-EBV(+) depending on PTLD diagnosis time.** The comparison of host-encoded miRNAs **(A)** miR-106a-5p, **(B)** miR-10a-5p, **(C)** miR-146b-5p, **(C)** miR-150-3p, **(E)** miR-21-5p, **(F)** miR-222-3p, **(G)** miR-23b-3b **(H)** miR-99a-5p expression in 10 PTLD-SOT-EBV(+) at 3 different timepoints (before, at and after diagnosis).


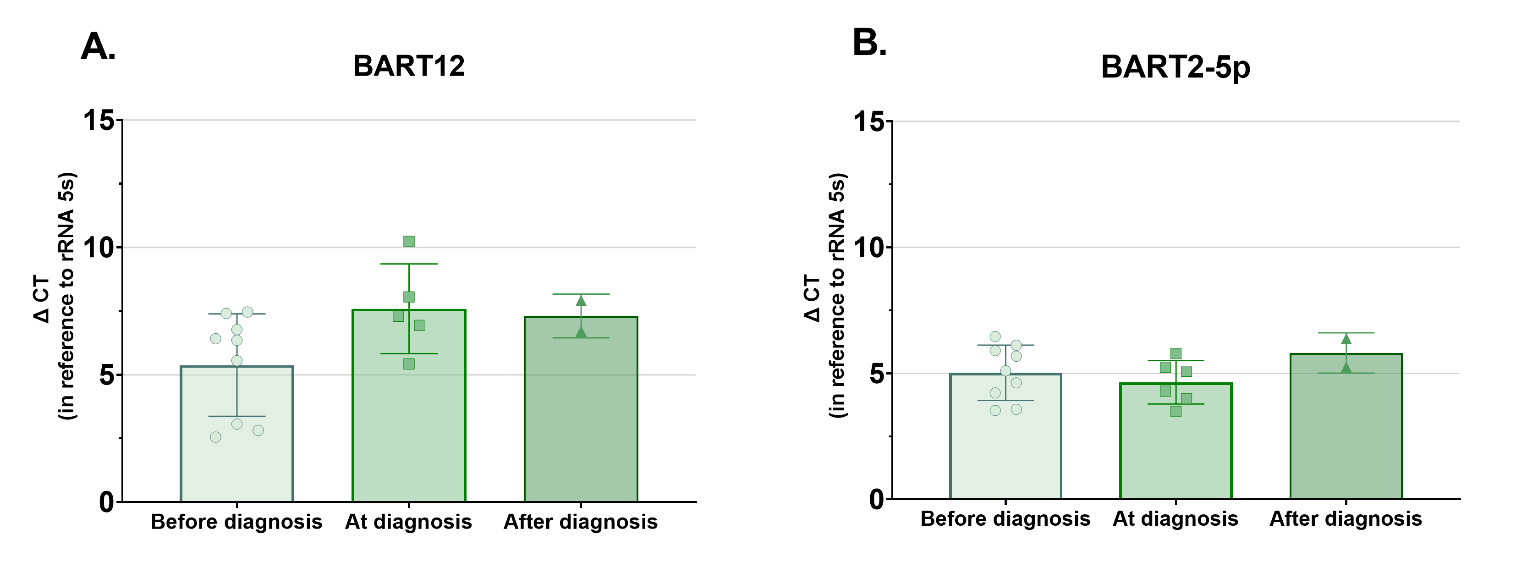
**Supplementary Figure 9. EBV-encoded miRNAs expression in PTLD-SOT-EBV(+) in function of PTLD diagnosis time.** The comparison of EBV-encoded miRNAs **(A)** BART12, **(B)** BART2-5p expression in 10 PTLD-SOT-EBV(+) at 3 different timepoints (before, at and after PTLD diagnosis).


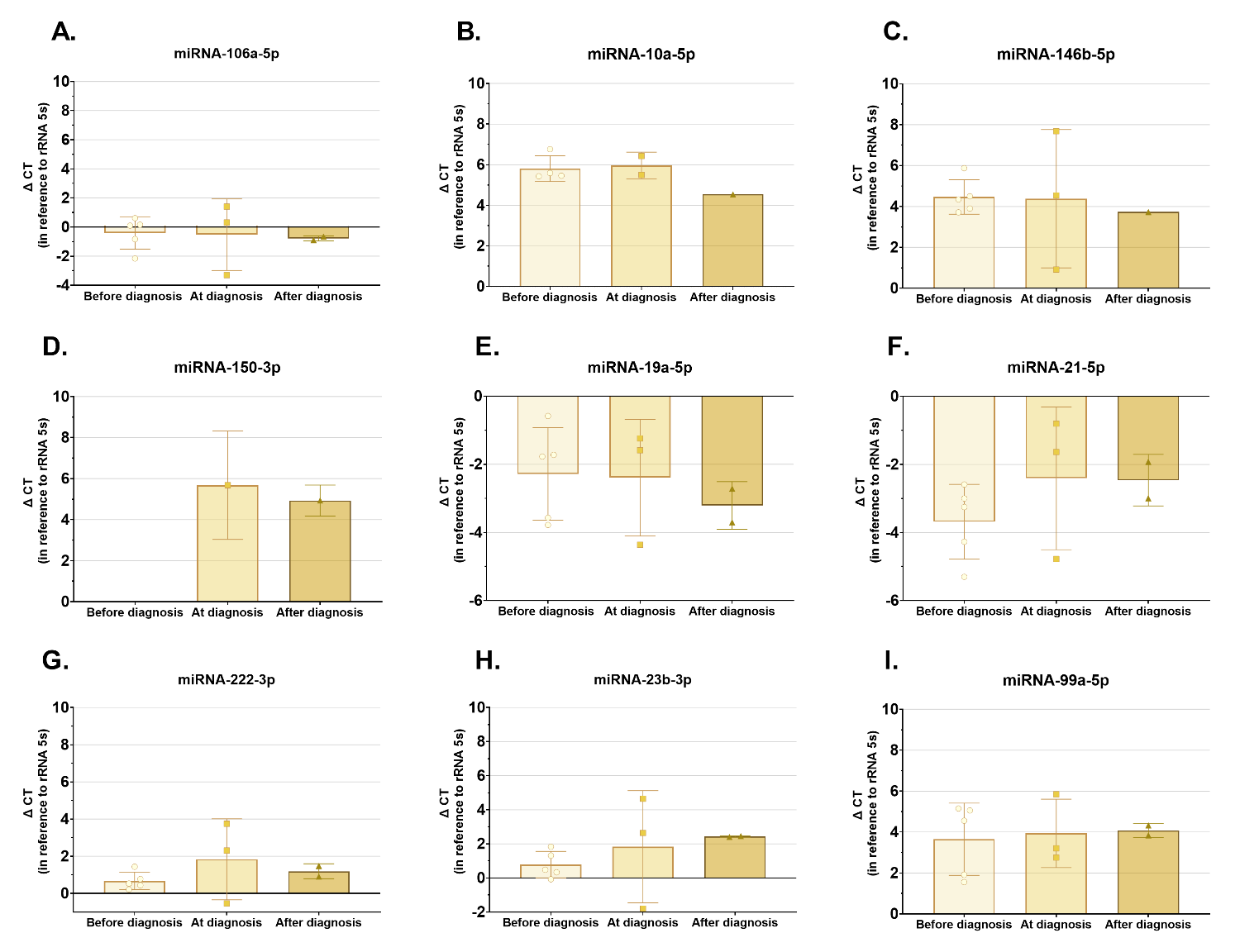
**Supplementary Figure 10. Host-encoded miRNAs expression in PTLD-SOT-EBV(-) in function of PTLD diagnosis time.** The comparison of host-encoded miRNAs **(A)** miR-106a-5p, **(B)** miR-10a-5p, **(C)** miR-146b-5p, **(D)** miR-150-3p, **(E)** miR-19a-5p, **(F)** miR-21-5p, **(G)** miR-222-3p, **(H)** miR-23b-3b **(I)** miR-99a-5p expression in 8 PTLD-SOT-EBV(-) at 3 different timepoints (before, at and after PTLD diagnosis).


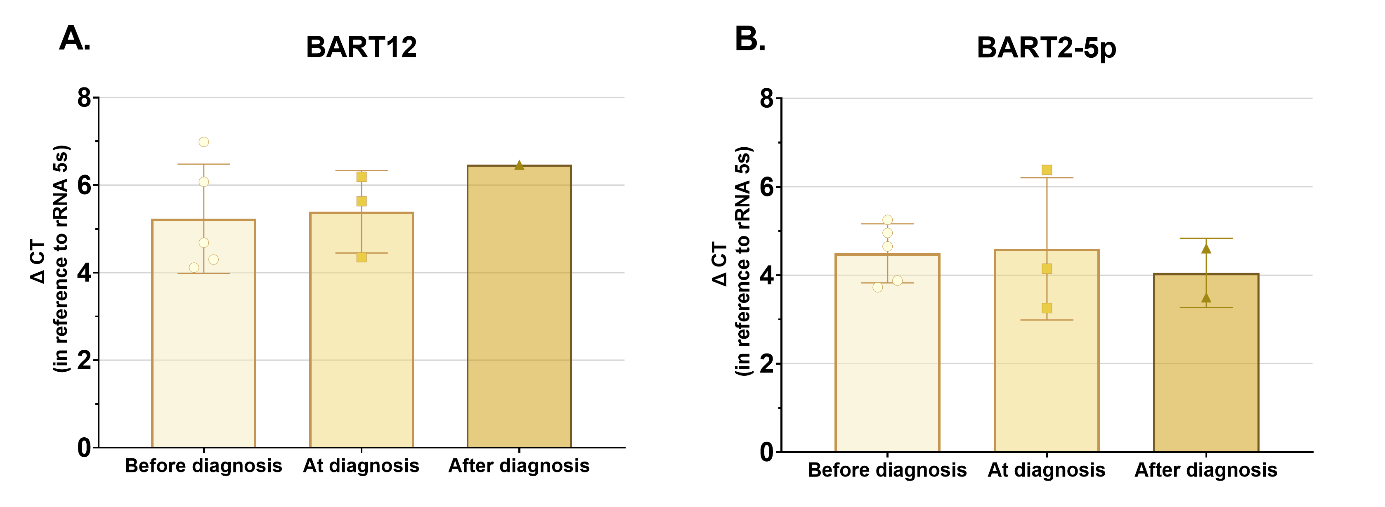


**Supplementary Figure 11. EBV-encoded miRNAs expression in PTLD-SOT-EBV(-) in function of diagnosis time.** The comparison of EBV-encoded miRNAs **(A)** BART12, **(B)** BART2-5p expression in 8 PTLD-SOT-EBV(-) at 3 different timepoints (before, at and after PTLD diagnosis).


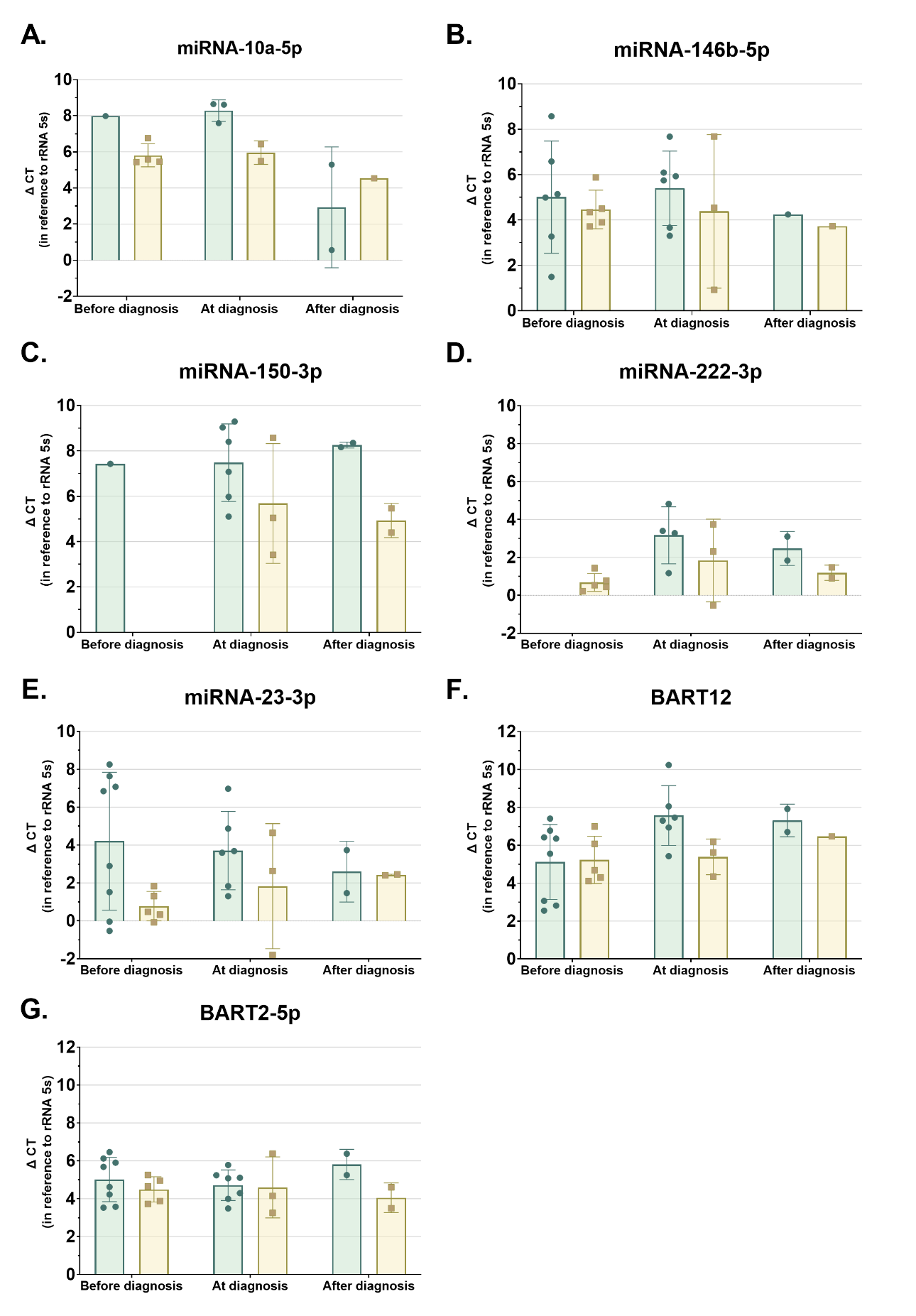
**Supplementary Figure 12. Exosomal miRNAs expression in PTLD-SOT-EBV(+) compared to PTLD-SOT-EBV(-) patients in function of PTLD diagnosis time.** The comparison of host-encoded miRNAs **(A)** miR-10a-5p, **(B)** miR-146b-5p, **(C)** miR-150-3p, **(D)** miR-222-3p, **(E)** miR-23-3p, and EBV-encoded **(F)** BART12 and **(G)** BART2-5p, in 10 PTLD-SOT-EBV(+) (green) and 8 PTLD-SOT-EBV(-) (yellow) at 3 different timepoints (before, at and after PTLD diagnosis).


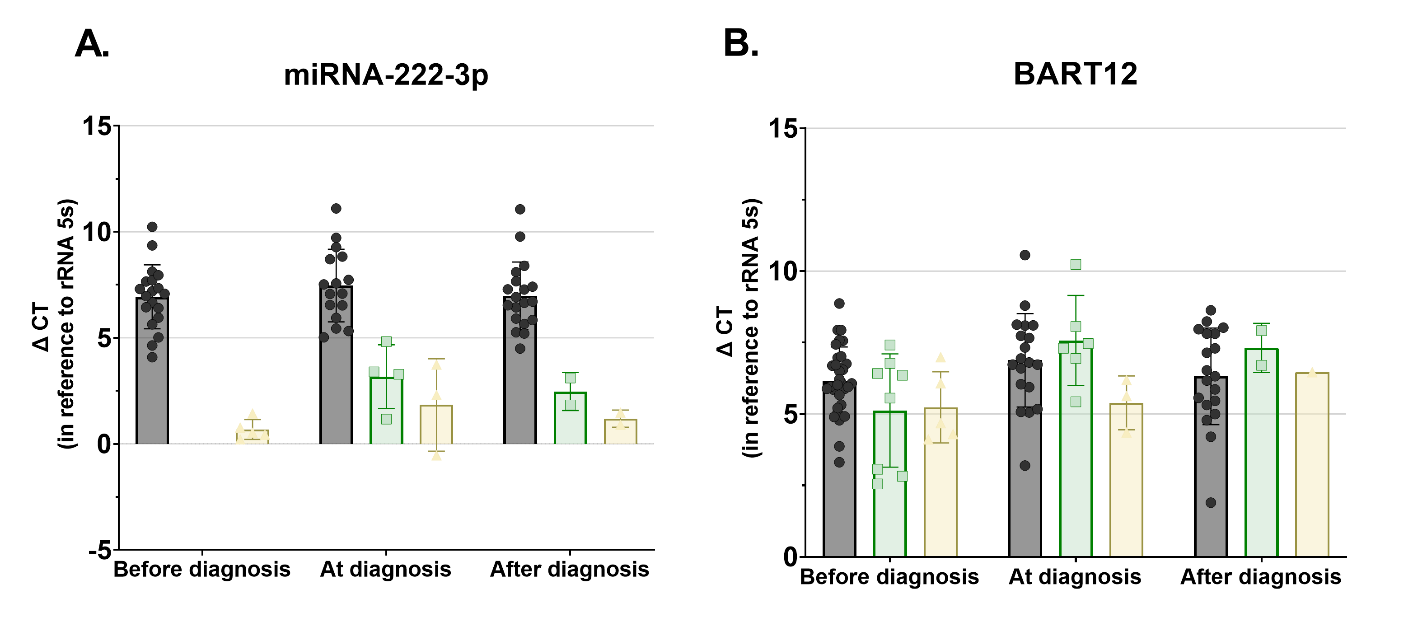


**Supplementary Figure 13. Comparison of miRNAs expression between PTLD patients after HSCT and SOT in function of PTLD diagnosis time.** The comparison of host-encoded **(A)** miR-222-3p, and EBV-encoded **(B)** BART12 expression in 23 PTLD-HSCT (black), 10 PTLD-SOT-EBV(+) (green) and in 8 PTLD-SOT-EBV(-) (yellow) at 3 different timepoints (before, at and after PTLD diagnosis).


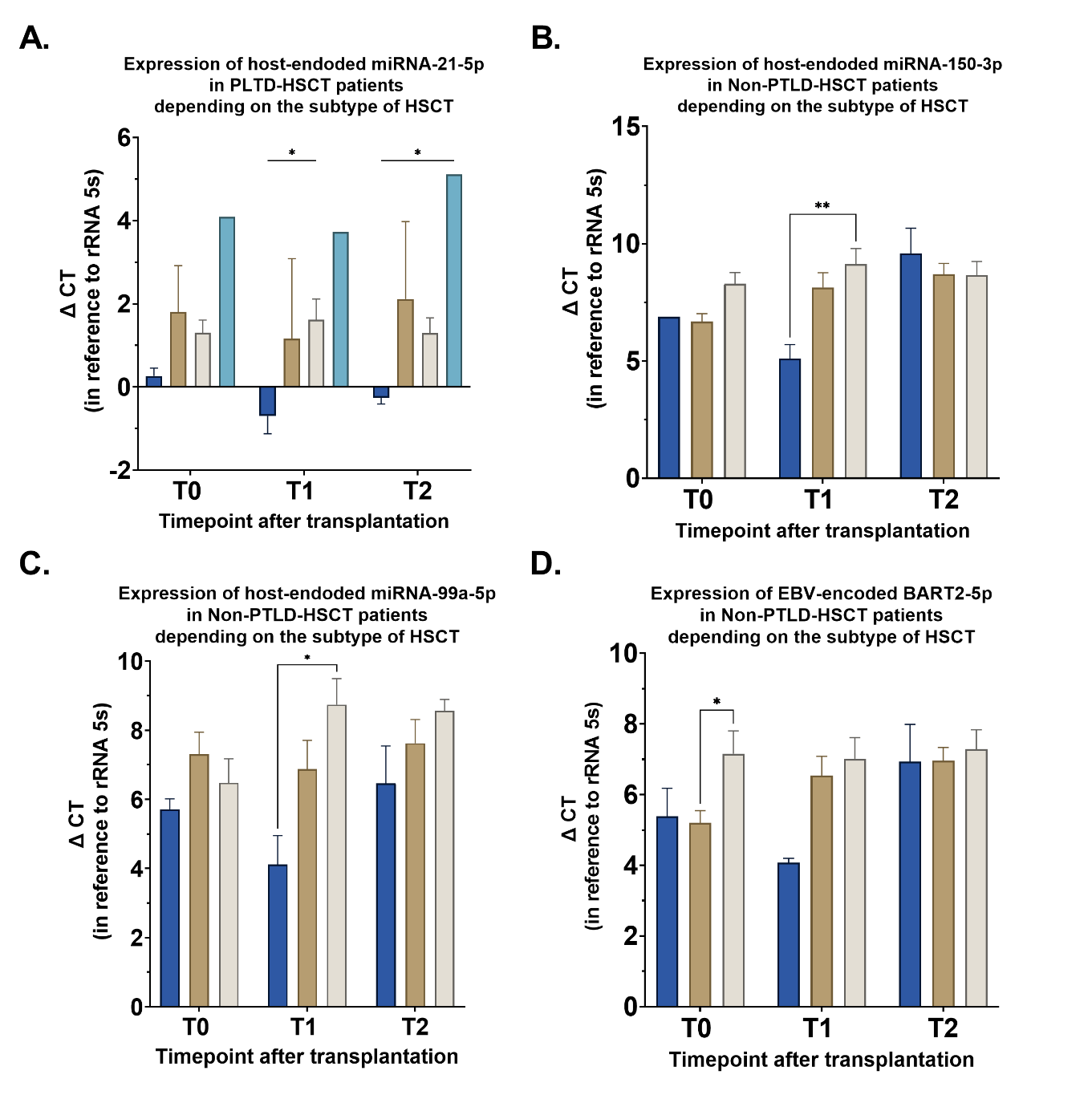


**Supplementary Figure 14. Correlation between miRNAs expression and clinical information in post-HSCT patients.** The correlation between the **(A)** expression of host-encoded miR-21-5p in PTLD-HSCT patients and the subtype of HSCT (haplo-identical [haplo-id] donor in dark blue, matched related donor [MRD] in brown, matched unrelated donor [MUD] in beige and umbilical cord blood [UCB] in teal) at T0,T1 and T2. Expression of host encoded **(B)** miR-150-3p, **(C)** miR-99a-5p and EBV-encoded **(D)** BART2-5p in Non-PTLD-HSCT patients depending on the subtype of HSCT (Haplo-id in dark blue, MRD in brown, MUD in beige) at 3 different timepoints. Significance was determined as a P value of <0.05 and SD is shown. (*) p ≤0.05, (**) p<0.01, (***) p<0.001, (****) p<0.0001.


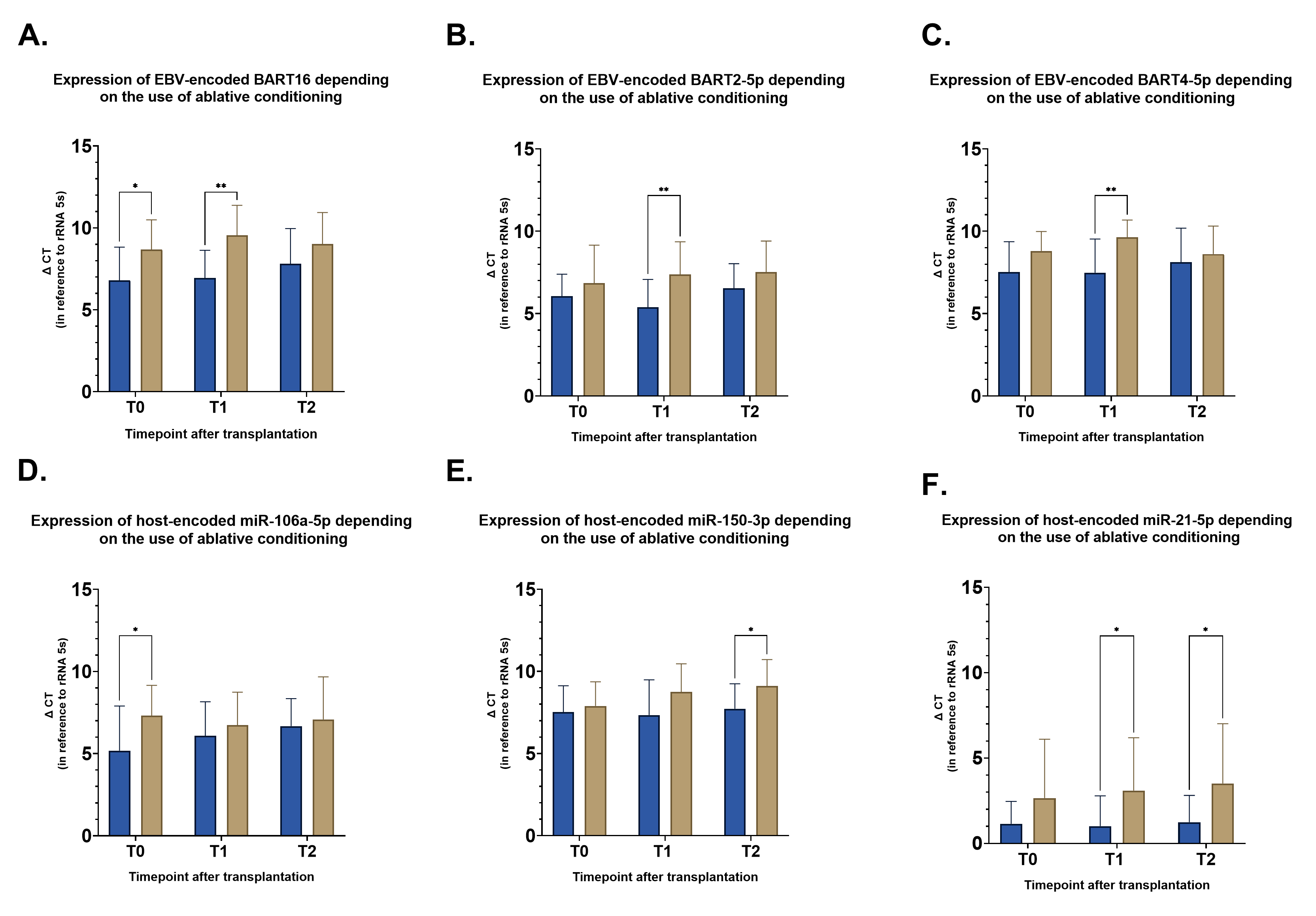


**Supplementary Figure 15. Correlation between miRNAs expression and ablative conditioning in post-HSCT patients.** The correlation between the expression of **(A)** BART16**, (B)**  BART2-5p, **(C)** BART4-5p, **(D)** miR-106a-5p, **(E)** miR-150-3p, **(F)** miR-21-5p in HSCT recipients who received ablative conditioning (blue) or did not receive ablative conditioning (brown) prior to HSCT, at 3 different timepoints. Significance was determined as a P value of <0.05 and SD is shown. (*) p ≤0.05, (**) p<0.01, (***) p<0.001, (****) p<0.0001.
